# Cholesterol Regulates Innate Immunity via Nuclear Hormone Receptor NHR-8

**DOI:** 10.1016/j.isci.2020.101068

**Published:** 2020-04-18

**Authors:** Benson Otarigho, Alejandro Aballay

**Affiliations:** 1 Department of Molecular Microbiology & Immunology, Oregon Health & Science University, Portland, OR 97239, USA

**Keywords:** Biochemistry, Immunity, Microbial Metabolism

## Abstract

Cholesterol is an essential nutrient for the function of diverse biological processes and for steroid biosynthesis across metazoans. However, the role of cholesterol in immune function remains understudied. Using the nematode *Caenorhabditis elegans*, which depends on the external environment for cholesterol, we studied the relationship between cholesterol and innate immunity*.* We found that the transporter CHUP-1 is required for the effect of cholesterol in the development of innate immunity and that the cholesterol-mediated immune response requires the nuclear hormone receptor NHR-8. Cholesterol acts through NHR-8 to transcriptionally regulate immune genes that are controlled by conserved immune pathways, including a p38/PMK-1 MAPK pathway, a DAF-2/DAF-16 insulin pathway, and an Nrf/SKN-1 pathway. Our results indicate that cholesterol plays a key role in the activation of conserved microbicidal pathways that are essential for survival against bacterial infections.

## Highlights

• Cholesterol is required for *C. elegans* immunity against *P. aeruginosa* infection
• Cholesterol is required during animal development for proper immunity and lifespan
• CHUP-1 is required for the effect of cholesterol in defense against infection
• Cholesterol acts through NHR-8 to transcriptionally regulate immune genes

## Graphical Abstract

**Figure:**
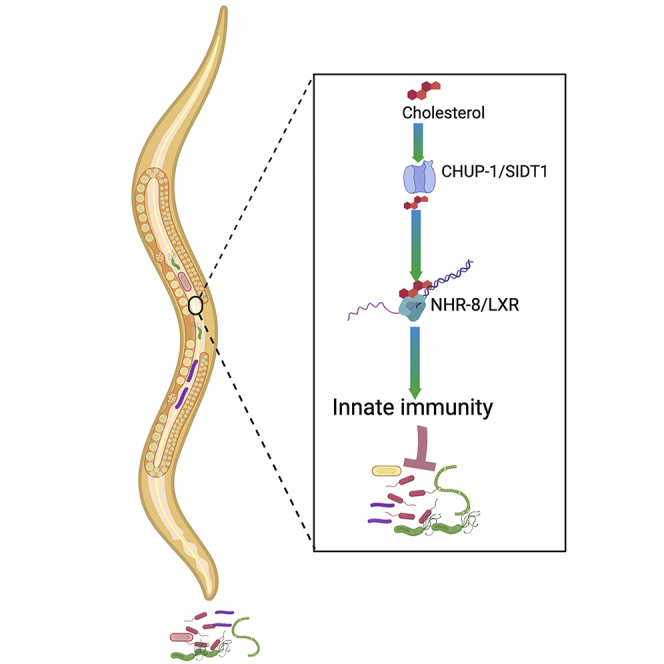

## Introduction

Cholesterol is an important nutrient and precursor for bile, vitamin D, oxysterols, and steroid hormones (Magner et al., 2013, Prabhu et al., 2016). In addition, it plays an important role in diverse biological processes, including metabolism, membrane structure, and cell signaling (Ihara et al., 2017, Kawasaki et al., 2013, Magner et al., 2013, Shanmugam et al., 2017), and has been implicated in the regulation of lifespan and aging (Chen et al., 2019, Cheong et al., 2011, Cheong et al., 2013, Ihara et al., 2017, Lee et al., 2005, Lee et al., 2007, Lee and Schroeder, 2012, Magner et al., 2013, Shanmugam et al., 2017). Despite this wealth of information about cholesterol's functions, there is little information about its role in the immune system, which is essential for defense against invading pathogens.

Vertebrates have a functional mevalonate pathway that is involved in the synthesis of cholesterol and other useful lipids that are synthesized from acetyl-CoA through the activity of the 3-hydroxy-3-methyl-glutaryl-coenzyme A reductase (Sapir et al., 2014). Although the mevalonate pathway is evolutionarily conserved in the nematode *Caenorhabditis elegans*, the animal lacks this reductase (Rauthan and Pilon, 2011). Thus, *C. elegans* depends on the external environment for cholesterol or other sterol supplements (Chitwood and Lusby, 1991, Hieb and Rothstein, 1968, Shanmugam et al., 2017). The possibility of tightly controlling cholesterol concentrations has facilitated the use of *C. elegans* to study cholesterol and lipid homeostasis to answer critical biological questions about health and longevity (Cheong et al., 2011, Cheong et al., 2013, Ihara et al., 2017, Magner et al., 2013, Shanmugam et al., 2017).

*C. elegans* imports cholesterol via conserved cholesterol transporters, such as CHUP-1 (cholesterol uptake associated) (Méndez-Acevedo et al., 2017, Valdes et al., 2012, Whangbo et al., 2017), NPC1 (Ikonen, 2008, Rosenbaum et al., 2009, Smith and Levitan, 2007), and other related proteins (Brown et al., 2008, Chen et al., 2016, Chen et al., 2019, Zhang et al., 2017). At the cellular level, different nuclear hormone receptors (NHRs), such as NHR-8, NHR-25, NHR-48, NHR-49, and NHR-80, bind and/or regulate cholesterol, lipids, hormone hemostasis, and metabolism in *C. elegans* (Antebi, 2006). In addition to their role in the coordination of metabolism, NHRs play key functions in the control of development, reproduction, and homeostasis (Bodofsky et al., 2017, Houthoofd et al., 2002, Piskacek et al., 2019, Ratnappan et al., 2016, Wang et al., 2015).

Here we investigated the relationship between cholesterol and innate immune defense in *C. elegans*. We found that cholesterol is required for proper development of immune defense against infection by the pathogen *Pseudomonas aeruginosa* and that the transporter CHUP-1 is required for the function of cholesterol in immunity. We also found that the cholesterol-mediated immune response requires NHR-8 to transcriptionally regulate immune genes that are controlled by conserved immune pathways, including a p38/PMK-1 MAPK pathway, a DAF-2/DAF-16 insulin pathway, and an Nrf/SKN-1 pathway. Our findings indicate that the innate immune system requires cholesterol to engage an NHR-8 immune pathway that primarily controls PMK-1 and is essential for host immune defense against pathogens.

## Results

### Cholesterol Is Required for *C. elegans* Defense against *P. aeruginosa* Infection

**Figure 1:**
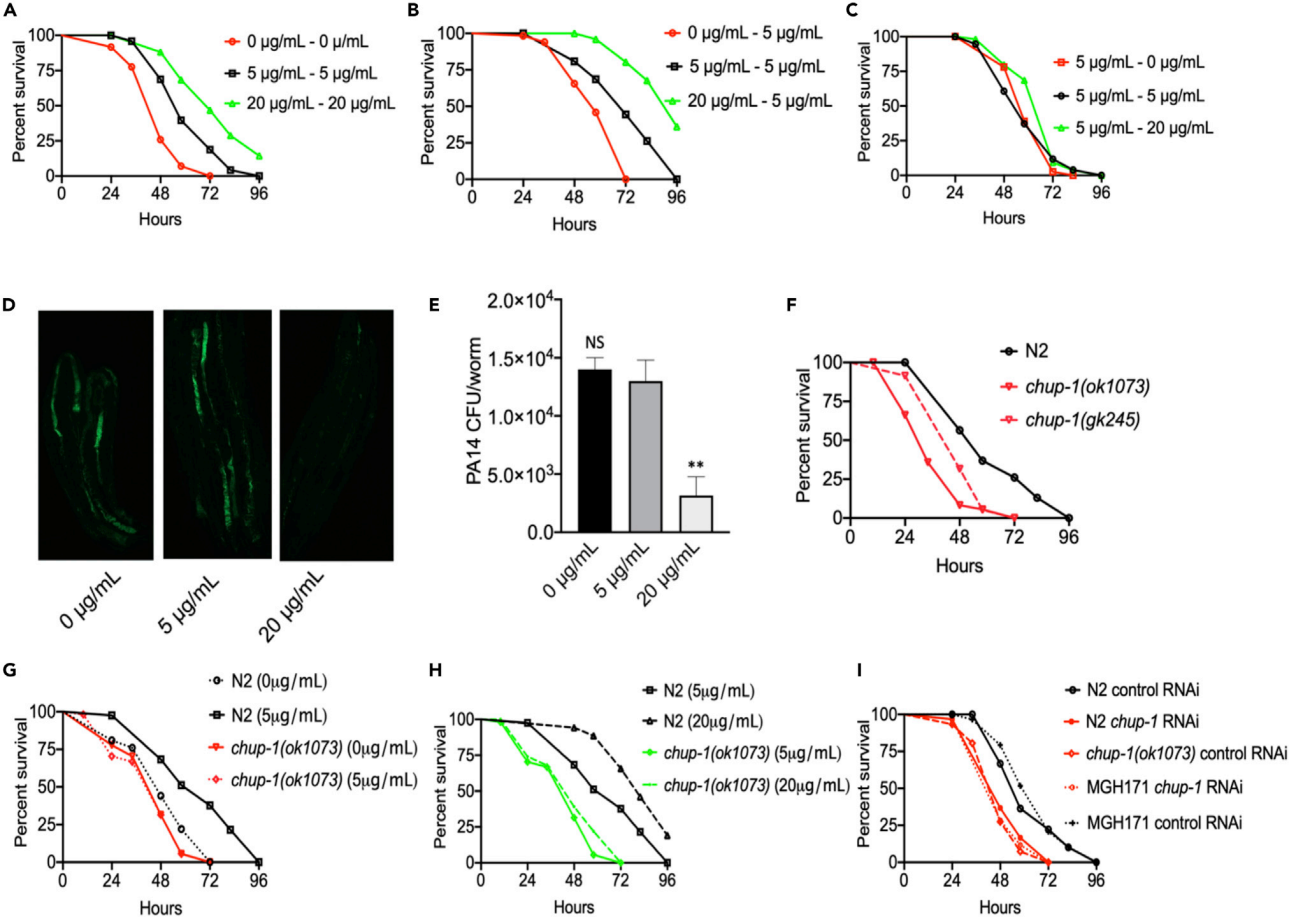
Cholesterol Is Required for *C. elegans* Resistance against *P. aeruginosa* (A) Wild-type animals were grown in the absence of cholesterol supplementation (0 μg/mL) or at different cholesterol concentrations, exposed to *P. aeruginosa* cultured on the same cholesterol concentrations, and scored for survival. WT animals grown on 5 μg/mL cholesterol (control) versus 0 μg/mL, P < 0.0001; 20 μg/mL, P < 0.0001. (B) WT animals were grown on different cholesterol concentrations, exposed to *P. aeruginosa* cultured at the control cholesterol concentration (5 μg/mL) and scored for survival. WT animals grown on 5 μg/mL cholesterol (control) versus 0 μg/mL, P < 0.0001; 20 μg/mL, P < 0.0001. (C) WT animals were grown on control cholesterol concentration (5 μg/mL), exposed to *P. aeruginosa* cultured on different cholesterol concentrations (0, 5 and 20 μg/mL) and scored for survival. WT animals grown on 5 μg/mL cholesterol (control) versus 0 μg/mL, P = NS; 20 μg/mL, P = NS. (D) WT animal colonization by *P. aeruginosa*-GFP after 24 h at 25°C. (E) Colony-forming units per animal grown on *P. aeruginosa* -GFP after 24 h at 25°C. Bars represent means, whereas error bars indicate SD; ∗∗p < 0.05, NS = not significant. (F) *chup-1(ok1073)* mutants were grown on 5 μg/mL cholesterol (control), exposed to *P. aeruginosa*, and scored for survival. WT animals versus *chup-1(ok1073)* and *chup-1(gk245),* P < 0.0001. (G) *chup-1(ok1073)* mutants were grown on 0 and 5 μg/mL cholesterol, exposed to *P. aeruginosa*, and scored for survival. WT animals grown on 5 μg/mL cholesterol (control) versus 0 μg/mL, P < 0.0001; *chup-1(ok1073)*, P < 0.0001. (H) *chup-1(ok1073)* mutants were grown on 20 and 5 μg/mL cholesterol, exposed to *P. aeruginosa*, and scored for survival. WT animals grown on 5 μg/mL cholesterol (control) versus 20 μg/mL, P < 0.0001; *chup-1(ok1073)* 20 μg/mL, P < 0.0001; *chup-1(ok1073)* 5 μg/mL, P < 0.0001. (I) Control, MGH171(*chup-1* RNAi), WT (*chup-1* RNAi) animals were grown on 5 μg/mL cholesterol, exposed to *P. aeruginosa*, and scored for survival. WT on control RNAi versus *chup-1(ok1073)* control RNAi, P < 0.0001; WT *chup-1* RNAi, P < 0.0001; MGH171 *chup-1* RNAi, P < 0.0001; MGH171 control RNAi, P = NS.

To study the role of cholesterol in innate immunity, we performed infections with the pathogen *P. aeruginosa* using wild-type *C. elegans* previously grown on lawns of *E. coli* in the absence of cholesterol supplementation; with 5 μg/mL cholesterol, which is the standard laboratory concentration to propagate the nematodes; and with 20 μg/mL cholesterol. Because strict sterol-free conditions affect the development of the animal, reduce the brood size, and result in dauer formation in the second generation (Matyash et al., 2004, Merris et al., 2003), in our studies we used conventional nematode growth media, which contains sufficient sterols to support *C. elegans* development. As shown in Figure S1, the absence or presence of cholesterol supplementation did not change the brood size or the development of the animals. To further address whether the absence of cholesterol supplementation affects the development of the animal, we used the GR1452 strain, which is a reporter of gene *col-19* that is sharply expressed at the late L4/young adult transition (Hayes et al., 2011). The absence of cholesterol supplementation did not affect the expression of GFP driven by the promoter of *col-19*, suggesting that the development of the animals under different cholesterol concentrations is similar (Figure S1C-D). Although GFP could accumulate over time and the results might vary when looked at specific time points before and after the molting, we did not observe any delay in the development of the animals into young adults (Figure S1A), which were used in our survival studies. We found that young adult animals grown in the absence of cholesterol supplementation were more susceptible to *P. aeruginosa-*mediated killing than animals grown in the presence of 5 μg/mL cholesterol (referred to as control cholesterol) (Figure 1A). In contrast, animals grown on plates containing 20 μg/mL cholesterol (referred to as high cholesterol) were more resistant to pathogen infection than animals grown on control plates (Figure 1A), indicating that cholesterol was required for *C. elegans* defense against *P. aeruginosa* infection.

To address the possibility that the absence of cholesterol supplementation might reduce the virulence of *P. aeruginosa,* we only changed the cholesterol concentrations prior to infection. As shown in Figure 1B, the presence or absence of cholesterol supplementation before infection affected the susceptibility of the animals to *P. aeruginosa*. Consistent with the idea that cholesterol acts on the host immune system rather than on *P. aeruginosa* virulence, there was no significant difference in the susceptibility of animals grown on standard cholesterol concentrations and animals infected with *P. aeruginosa* grown on different cholesterol concentrations (Figure 1C). The effect of cholesterol-mediated immune defense on *P. aeruginosa* bacterial colonization was examined by visualizing bacteria expressing GFP and quantifying the number of bacterial cells in the intestine of the animals. Although the absence of cholesterol supplementation had no effect on bacterial burden, high cholesterol significantly reduced it (Figures 1D and 1E). Taken together, our findings indicate that cholesterol is required during the development of *C. elegans* for proper resistance against *P. aeruginosa* infection.

Because several studies have indicated that cholesterol is critical for *C. elegans* lifespan (Cheong et al., 2011, Cheong et al., 2013, Ihara et al., 2017, Lee et al., 2005, Lee and Schroeder, 2012, Magner et al., 2013, Shanmugam et al., 2017), we studied the effect of different cholesterol concentrations on the survival of animals grown on control *E. coli*. Although animals grown in the absence of cholesterol supplementation exhibited a shorter lifespan, those grown on high cholesterol exhibited a longer lifespan than control animals (Figure S2A). Because *E. coli* proliferation is a cause of death in *C. elegans* (Garigan et al., 2002, Sutphin and Kaeberlein, 2009) and animals deficient in the immune response are persistently colonized and killed by *E. coli* (Kerry et al., 2006, Singh and Aballay, 2006, Tenor and Aballay, 2008), we examined the effect of cholesterol on the lifespan of animals on lawns of heat-killed *E. coli.* The survival of animals grown in the absence of cholesterol supplementation was not significantly different from that of control animals, indicating that the absence of cholesterol supplementation did not significantly affect lifespan. In contrast, the animals that were grown on high cholesterol had a slightly longer lifespan than the control animals (Figure S2B). These findings indicate that cholesterol is required for defense against infection and that cholesterol supplementation during animal development may not only boost the immune response but also improve the lifespan of the animals.

*C. elegans* is auxotrophic for cholesterol (Chitwood and Lusby, 1991, Hieb and Rothstein, 1968) and requires different proteins to bind and transport cholesterol (Brown et al., 2008, Chen et al., 2016, Chen et al., 2019, Huber et al., 2006, Kamal et al., 2019, Méndez-Acevedo et al., 2017, Ranawade et al., 2018, Sym et al., 2000, Valdes et al., 2012, Zhang et al., 2017). Thus, we investigated the roles of different transporters in the cholesterol-mediated immune response by exposing loss-of-function mutants in genes known to encode cholesterol transporters to *P. aeruginosa.* We found that animals carrying deletions in genes *chup-1* and *sms-5* showed enhanced susceptibility to *P. aeruginosa-*mediated killing at control cholesterol concentrations (Figures 1F, 1G, and S3). However, only *chup-1* mutation suppressed the enhanced resistance to *P. aeruginosa-*mediated killing induced by high cholesterol (Figure 1H), suggesting that CHUP*-*1 is the only required transporter for the effect of high cholesterol supplementation on pathogen resistance. The intestinal contribution of CHUP-1 in cholesterol-mediated defense was examined by utilizing a *C. elegans* strain capable of RNAi activity only in the intestine (strain MGH171), in which RNAi knockdown of CHUP-1 completely suppressed the effect of high cholesterol on *C. elegans* resistance to *P. aeruginosa* infection (Figures 1I, S3C, and S3D). These results suggest that CHUP-1 is required in the intestine to mediate the effect of cholesterol on pathogen resistance.

### Transcriptomics Identification of Cholesterol-Dependent Immune Genes

**Figure 2:**
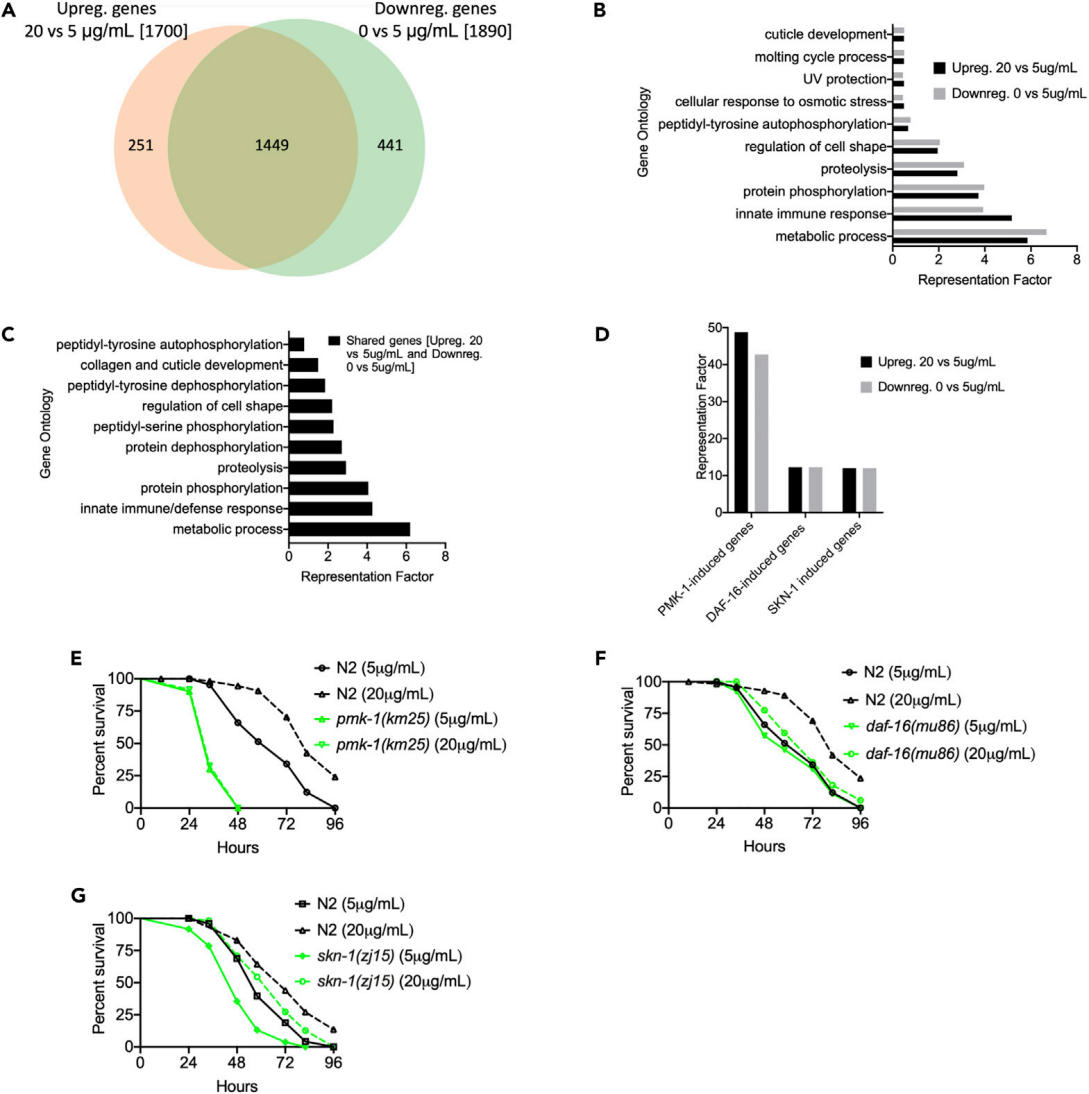
Cholesterol-Mediated Immunity Primarily Acts through a p38/PMK-1 MAPK Pathway (A) Venn diagram showing upregulated genes (20 versus 5 μg/mL cholesterol) and downregulated genes (0 versus 5 μg/mL cholesterol). (B) Gene ontology analysis of upregulated and downregulated genes in animals grown on 20 and 0 μg/mL cholesterol, respectively. The cutoff is based on the filtering thresholds of P < 0.05 and arranged according to the representation factor. (C) Gene ontology analysis of shared genes between animals grown at 20 versus 5 μg/mL and 0 versus 5 μg/mL cholesterol. The cutoff is based on the filtering thresholds of P < 0.05 and arranged according to representation factor. (D) Representation factors of immune pathways for the upregulated and downregulated immune genes in animals grown at 20 versus 5 and 0 versus 5 μg/mL cholesterol, respectively. (E) WT and *pmk-1(km25)* animals were grown on 20 and 5 μg/mL cholesterol, exposed to *P. aeruginosa*, and scored for survival. WT animals grown on 5 μg/mL cholesterol (control) versus 20 μg/mL, P < 0.0001; *pmk-1(km25)* 5 μg/mL, P < 0.0001; *pmk-1(km25)* 20 μg/mL, P > 0.0001. *pmk-1(km25)* 0 μg/mL versus *pmk-1(km25)* 5 μg/mL, P = NS. (F) WT and *daf-16(mu86)* animals were grown on 20 and 5 μg/mL cholesterol, exposed to *P. aeruginosa*, and scored for survival. WT animals grown on 5 μg/mL cholesterol (control) versus 20 μg/mL, P < 0.0001; *daf-16(mu86)* 20 μg/mL, P = NS; *daf-16(mu86)* 5 μg/mL, P = NS. (G) WT and *skn-1(zj15)* animals were grown on 20 and 5 μg/mL cholesterol, exposed to *P. aeruginosa*, and scored for survival. *skn-1(zj15)* animals grown on 5 μg/mL cholesterol (control) versus 20 μg/mL, P < 0.001.

To gain insights into the host defense mechanisms that require cholesterol to combat bacterial infections, we performed transcriptomics analyses to identify genes that were upregulated in animals grown on high cholesterol or downregulated in animals grown in the absence of cholesterol supplementation relative to animals grown on the control cholesterol concentration (Table S1). Overall, the gene expression data showed an important overlap between genes that were upregulated by high cholesterol and those that were downregulated in the absence of cholesterol supplementation (Figure 2A). To identify related gene groups that were responsible for the effect of cholesterol on resistance to *P. aeruginosa* infection, we employed an unbiased gene enrichment analysis using the database for annotation, visualization, and integrated discovery (DAVID, http://david.abcc.ncifcrf.gov) (Dennis et al., 2003) (Table S2). The 10 Gene Ontology (GO) clusters with the highest DAVID enrichment score for a number of vital biological functions are shown in Figure 2B. For the subset of genes that were upregulated in animals grown on high cholesterol or downregulated in animals grown in the absence of cholesterol supplementation, the metabolic process cluster was the most highly enriched, followed by the innate immune/defense cluster (Figure 2B and Table S3). As expected, a similar enrichment was also observed using a Wormbase enrichment analysis tool (https://wormbase.org/tools/enrichment/tea/tea.cgi) (Angeles-Albores et al., 2016, Angeles-Albores et al., 2018) that is specific for *C. elegans* gene data analyses (Figures S4A and S4B). Metabolic and immune genes were also highly enriched among the 1,449 genes that overlapped (Figures 2C and S4B).

We used WormExp (http://wormexp.zoologie.uni-kiel.de/wormexp/) (Yang et al., 2016), which integrates all published expression data for *C. elegans*, to analyze the two most highly enriched GO clusters. The further analysis of the metabolic cluster revealed a number of genes that are differentially expressed during development (Table S4), which suggest that, even though animals fed different cholesterol concentrations seem to reach adulthood at the same time (Figure S1A), there may be small differences during larval development among the different populations used in this study. The study of the immune cluster indicated that genes controlled by a p38/PMK-1 MAPK pathway were the most highly overrepresented among those upregulated by high cholesterol or downregulated by the absence of cholesterol supplementation (Tables S4 and S5). Other pathways involved in *C. elegans* innate immunity, including a DAF-2/DAF-16 insulin pathway and a Nrf/SKN-1, were also enriched (Figure 2D and Table S5). Several of the SKN-1-, ELT-2-, DAF-16-dependent genes are also controlled by PMK-1 (Tables S4 and S5), indicating that the PMK-1 pathway is a main pathway by which high cholesterol promotes innate immunity. The activation of PMK-1 by high cholesterol was confirmed by directly measuring the levels of active PMK-1 (Figure S4C). Taken together, these results indicate that cholesterol enhances *C. elegans* resistance to *P. aeruginosa* mainly by activating immune genes, several of which are controlled by the PMK-1 immune pathway.

We hypothesized that the enhanced resistance to *P. aeruginosa* infection of animals grown on high cholesterol might be due to the upregulation of immune genes. To test this hypothesis, we studied the role of suppression by mutation or RNAi of the immune pathways transcriptionally regulated by cholesterol. As shown in Figures 2E–2G, inactivation of *pmk-1*, *daf-16,* and *skn-1* completely or partially suppressed the enhanced resistance to *P. aeruginosa* induced by high cholesterol. Inhibition of *pmk-1* or *daf-16* did not enhance the effect of the absence of cholesterol supplementation (Figures S5A and S5B), further confirming that the cholesterol effect on animal survival following *P. aeruginosa* infection was due to the regulation of immune pathways. To address whether additional genes crucial for immunity are generally required for the beneficial effects of high cholesterol on defense against infection, we used *kgb-1* and *dbl-1* mutants, which have been shown to be susceptible to *P. aeruginosa* infection (Evans et al., 2008, Mallo et al., 2002, Pellegrino et al., 2014). Even though these animals are susceptible to *P. aeruginosa*-mediated killing, high cholesterol was able to improve their survival (Figures S5C–S5F). Consistent with the observed gene enrichment in PMK-1-dependent genes, these results indicate that PMK-1, and partially DAF-16 and SKN-1, are required for the immune activation caused by the presence of high cholesterol.

### Cholesterol Functions through NHR-8/PMK-1 to Promote Innate Immunity

**Figure 3:**
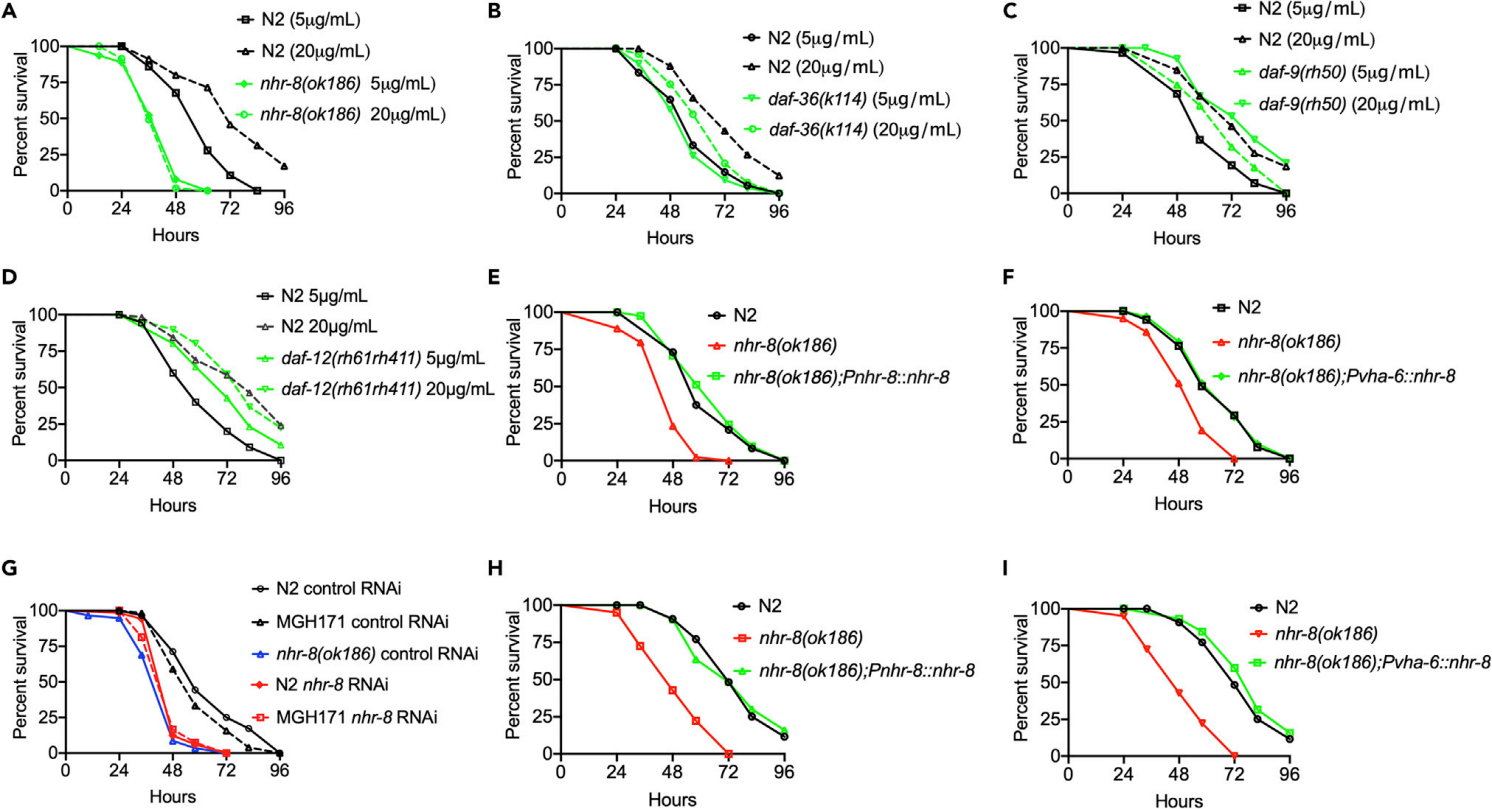
The Nuclear Hormone Receptor NHR-8 Mediates the Cholesterol Effect on the Immune System (A) WT and *nhr-8(ok186)* animals were grown on 20 and 5 μg/mL cholesterol, exposed to *P. aeruginosa*, and scored for survival. WT animals grown on 5 μg/mL cholesterol (control) versus WT animals 20 μg/mL, P < 0.0001; *nhr-8(ok186)* 20 μg/mL, P < 0.0001; *nhr-8(ok186)* 5 μg/mL, P < 0.0001. *nhr-8(ok186)* mutant on 20 μg/mL versus *nhr-8(ok186)* 5 μg/mL, P = NS. (B) WT and *daf-36(k114)* animals were grown on 20 and 5 μg/mL cholesterol, exposed to *P. aeruginosa*, and scored for survival. WT animals grown on 5 μg/mL cholesterol (control) versus WT animals 20 μg/mL, P < 0.0001; *daf-36(k114)* 20 μg/mL, P < 0.0001; *daf-36(k114)* 5 μg/mL, P < 0.0001. *daf-36(k114)* animals on 20 μg/mL versus *daf-36(k114)* 5 μg/mL, P < 0.0001. (C) WT and *daf-9(rh50)* animals were grown on 20 and 5 μg/mL cholesterol, exposed to *P. aeruginosa*, and scored for survival. WT animals grown on 5 μg/mL cholesterol (control) versus WT animals 20 μg/mL, P < 0.0001; *daf-9(rh50)* 20 μg/mL, P < 0.0001; *daf-9(rh50)* 5 μg/mL, P < 0.0001. *daf-9(rh50)* animals on 20 μg/mL versus *daf-9(rh50)* 5 μg/mL, P < 0.0001. (D) WT and *daf-12(rh61rh411)* animals were grown on 20 and 5 μg/mL cholesterol, exposed to *P. aeruginosa*, and scored for survival. WT animals grown on 5 μg/mL cholesterol (control) versus WT animals 20 μg/mL, P < 0.0001; *daf-12(rh61rh411)* 20 μg/mL, P < 0.0001; *daf-12(rh61rh411)* 5 μg/mL, P < 0.0001. *daf-12(rh61rh411)* animals on 20 μg/mL versus *daf-12(rh61rh411)* 5 μg/mL, P < 0.0001. (E) WT, *nhr-8(ok186)*, and *nhr-8(ok186);Pnhr-8::nhr-8* animals were grown on 5 μg/mL cholesterol, exposed to *P. aeruginosa*, and scored for survival. WT animals versus *nhr-8(ok186),* P < 0.0001; *nhr-8(ok186);Pnhr-8::nhr-8*, P = NS. (F) WT, *nhr-8(ok186)*, and *nhr-8(ok186);Pvha-6::nhr-8* animals were grown on 5 μg/mL cholesterol, exposed to *P. aeruginosa,* and scored for survival. WT animals versus *nhr-8(ok186),* P < 0.0001; *nhr-8(ok186);Pvha-6::nhr-8*, P = NS. (G) Control, *nhr-8* RNAi WT and MGH171 animals were grown on 5 μg/mL cholesterol, exposed to *P. aeruginosa,* and scored for survival. WT control RNAi versus MGH171 *nhr-8* RNAi, P < 0.0001. (H) WT, *nhr-8(ok186)*, and *nhr-8(ok186);Pnhr-8::nhr-8* animals were grown on 20 μg/mL cholesterol, exposed to *P. aeruginosa*, and scored for survival. WT animals versus *nhr-8(ok186),* P < 0.0001; *nhr-8(ok186);Pnhr-8::nhr-8*, P = NS. (I) WT, *nhr-8(ok186)*, and *nhr-8(ok186);Pvha-6::nhr-8* animals were grown on 20 μg/mL cholesterol, exposed to *P. aeruginosa,* and scored for survival. WT animals versus *nhr-8(ok186),* P < 0.0001; *nhr-8(ok186);Pvha-6::nhr-8*, P = NS.

Because cholesterol is a required precursor for the steroid biosynthesis pathway, which is evolutionarily conserved from *C. elegans* to mammals (Antebi, 2015, Calkin and Tontonoz, 2012, Fischer et al., 2013, Mooijaart et al., 2005, Rauthan and Pilon, 2011, Shanmugam et al., 2017, Watts and Ristow, 2017, Wollam et al., 2011, Yoshiyama-Yanagawa et al., 2011), we reasoned that cholesterol-derived steroids might be required for proper function of the innate immune system. *C. elegans* NHR-8 regulates a steroid biosynthesis pathway (Figure S6) that has been linked to the control of cholesterol balance, fatty acid desaturation, apolipoprotein production, bile acid metabolism, and xenobiotic metabolism (Chow et al., 2014, Lindblom and Dodd, 2006, Magner et al., 2013, Ménez et al., 2019, Schindler et al., 2014). Thus, we studied the susceptibility to *P. aeruginosa-*mediated killing of mutants in the known NHR-8 steroid biosynthesis pathways and found that only *nhr-8* mutant animals were more susceptible to *P. aeruginosa* compared with wild-type animals (Figures S7A–S7F). In addition, *nhr-8(ok186)* fully suppressed the beneficial effect of high cholesterol (Figure 3A). Similar results were obtained using the *nhr-8(tm1800)* null allele (Figure S7B). Unlike mutations in *nhr-8*, mutations in *daf-36*, *daf-9*, and *daf-12* did not result in enhanced susceptibility to *P. aeruginosa* infection under high or control cholesterol concentrations (Figures 3B–3D), indicating that they are not part of the cholesterol-induced NHR-8/PMK-1 pathway that promotes innate immunity.

Expression of *nhr-8* under the control of its own promoter fully rescued the mutant phenotype of *nhr-8(ok186)* (Figure 3E). Consistent with its function in the intestine, NHR-8 expression under the regulation of the intestine-specific promoter *Pvhp-6* also fully rescued the mutant phenotype of *nhr-8(ok186)* animals (Figure 3F). The intestinal function of NHR-8 in immunity was further confirmed using strain MGH171, which allows intestine-specific RNAi (Figure 3G). Expression of *nhr-8* under the control of its own promoter or *Pvhp-6* fully rescued the enhanced susceptibility to *P. aeruginosa* of *nhr-8(ok186)* animals grown on a high cholesterol concentration (Figures 3H and 3I). Overexpression of *nhr-8* in wild-type animals resulted in higher resistance to *P. aeruginosa* infection compared with control animals only when the animals were grown at 20 μg/mL cholesterol (Figure S8A), indicating that NHR-8 is a rate-limiting factor that mediates the enhanced immunity elicited by high cholesterol.

**Figure 4:**
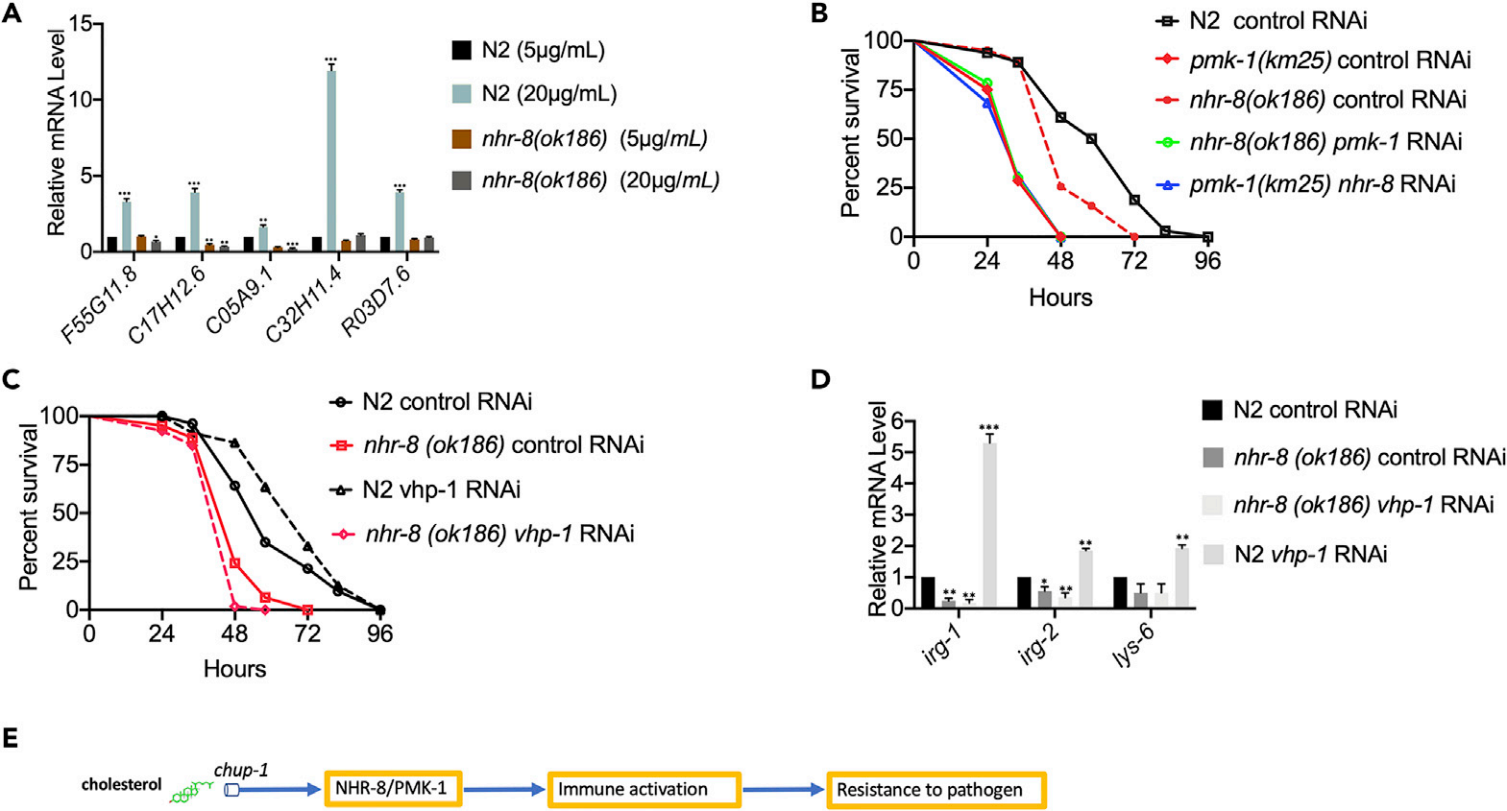
Cholesterol Activates the NHR-8/PMK-1 Immune Pathway (A) Expression of cholesterol-mediated immune genes in WT and *nhr-8(ok186)* animals grown on 5 and 20 μg/mL cholesterol. *pmk-1*- (*F55G11.8, C17H12.6, C05A9.1, C32H11.4* and *R03D7.6*) and *daf-16*-dependent genes (*F55G11.8, C05A9.1*) were studied. Bars represent means, whereas error bars indicate SD; ∗p < 0.05, ∗∗p < 0.001 and ∗∗∗p < 0.0001. (B) Control, *pmk-1(km25)*, *nhr-8(ok186)*, and WT treated with *pmk-1* RNAi and *nhr-8* RNAi were grown on 5 μg/mL cholesterol, exposed to *P. aeruginosa*, and scored for survival. WT animals control RNAi versus *pmk-1(km25)* control RNAi, P < 0.0001; *nhr-8(ok186)* control RNAi, P < 0.001; WT *pmk-1* RNAi, P < 0.0001; WT *nhr-8* RNAi, P < 0.0001; *nhr-8(ok186) pmk-1* RNAi, P < 0.0001. (C) Control, *nhr-8(ok186)*, and *vhp-1* RNAi were grown on 5 μg/mL cholesterol, exposed to *P. aeruginosa*, and scored for survival. WT animal versus *nhr-8(ok186)*, P < 0.001; *nhr-8(ok186) vhp-1* RNAi, P < 0.0001; N2 *vhp-1* RNAi, P < 0.001. (D) Gene expression of *nhr-8(ok186)* and WT animals with control or *vhp-1* RNAi grown on 5 μg/mL cholesterol. Bars represent means, whereas error bars indicate SD; ∗p < 0.05, ∗∗p < 0.001, and ∗∗∗p < 0.0001. (E) Model for activation of the NHR-8/PMK-1/p38 MAPK immune pathway by cholesterol.

The cholesterol effect on immune defense required intestinal NHR-8 (Figures 3F, 3G, and 3I), and animals grown on high cholesterol exhibited high expression levels of immune genes (Figures 2B–2D). Therefore, we hypothesized that mutation in *nhr-8* would suppress the upregulation of immune genes induced by high cholesterol. To test this hypothesis, we compared the gene expression of *nhr-8(ok186)* and wild-type animals grown on high cholesterol. We used *pmk-1*-dependent genes (*F55G11.8, C17H12.6, C05A9.1, C32H11.4,* and *R03D7.6*) that are cholesterol dependent (Table S1) and are known markers of innate immune activation in *C. elegans* (Ooi et al., 2012, Troemel et al., 2006). As shown in Figure 4A, *nhr-8* mutation suppressed the upregulation of PMK-1- and DAF-16-dependent genes elicited by high cholesterol. Although NHR-8 seemed to be required for the expression of PMK-1-dependent genes, it did not transcriptionally control *pmk-1* itself (Figure S9). Consistent with the idea that both NHR-8 and PMK-1 are part of the same immune pathway induced by cholesterol, *nhr-8* and *pmk-1* inactivation had no additive effect on the susceptibility of the animals to *P. aeruginosa-*mediated killing (Figure 4B). We observed no additive effect of inhibition by RNAi of the cholesterol transporter CHUP-1 on *nhr-8(ok186)* animals (Figure S8B), which provides additional support of the idea that NHR-8 is part of the pathway involved in the activation of PMK-1 by cholesterol.

To further substantiate the relationship between NHR-8 and the PMK-1 pathway, we studied whether *nhr-8* mutation could suppress the effect of *vhp-1* RNAi on the susceptibility of the animals to *P. aeruginosa*. VHP-1 inhibition by RNAi is known to promote the activation of PMK-1, which results in enhanced resistance to pathogen infection (Kim et al., 2004, Mizuno et al., 2004). The *nhr-8* mutation suppressed the enhanced resistance to *P. aeruginosa-*mediated killing and the enhanced gene expression of *vhp-1* RNAi animals (Figures 4C and 4D). Taken together, these studies show that cholesterol regulates innate immune defense against *P. aeruginosa* infection via an NHR-8/PMK-1 pathway (Figure 4E).

## Discussion

The host immune system fights infecting microbial pathogens through diverse molecular pathways (Akira et al., 2006, Keshet et al., 2017, Sun et al., 2011). The activation and function of these pathways depend on a myriad of genetic, environmental, and nutritional factors. Although the role of cholesterol in various physiological processes in animals is well studied (Ihara et al., 2017, Kawasaki et al., 2013, Magner et al., 2013, Shanmugam et al., 2017), its specific role in the function of the immune system during responses to infections is unknown. In this study, we uncovered the underlying mechanism of the cholesterol requirement for proper innate immune function in *C. elegans*. We further established that the cholesterol transporter CHUP-1 and the nuclear hormone receptor NHR-8 are required for the effect of cholesterol on pathogen resistance. In addition, we provided evidence indicating that cholesterol is required for the activation of immune pathways.

CHUP-1 is evolutionarily conserved, and SIDt1 and SIDt2 are 9-transmembrane domain transporters that are more closely related in humans (Méndez-Acevedo et al., 2017, Valdes et al., 2012, Whangbo et al., 2017). Although SIDTs transport both double-stranded RNA (dsRNA) and cholesterol (Whangbo et al., 2017), dsRNA transport cannot be attained without cholesterol (Valdes et al., 2012). Once inside *C. elegans* cells, cholesterol binds and activates NHR-8 (Motola et al., 2006, Rauthan and Pilon, 2011, Rottiers et al., 2006, Shanmugam et al., 2017, Watts and Ristow, 2017, Wollam et al., 2011, Yoshiyama-Yanagawa et al., 2011), which is also evolutionarily conserved (Hoffmann and Partridge, 2015, Magner et al., 2013, Ménez et al., 2019). Like most NHRs, *nhr-8* is a transcriptional regulator that is involved in steroid biosynthesis and metabolism homeostasis. Consistent with the role of cholesterol in steroid biosynthesis and metabolism, it is not surprising that genes involved in metabolic processes were among the most highly enriched genes, demonstrating altered expression when the animals were grown on different cholesterol concentrations.

Although the relationship between different nutrients and the proper function of the immune system has been studied in *C. elegans* (Komura et al., 2012, Shivers et al., 2009, Wu et al., 2019), the role of cholesterol has not been explored. The present analysis reveals evolutionarily conserved mechanisms that explain the role of cholesterol for proper innate immune activation against invading *P. aeruginosa*, in which a known transporter, CHUP-1, is required for the effect of cholesterol on pathogen resistance. The cholesterol effect on the immune system is mediated by the expression of immune genes that are primarily controlled by the PMK-1 pathway, some of which are also controlled by DAF-16 and SKN-1. The results also indicate that cholesterol activation of PMK-1-dependent gene expression requires NHR-8. The results presented here provide a better understanding of how cholesterol plays a role in the elicitation of innate immunity via conserved pathways that protect the host against pathogenic bacteria.

### Limitation of the Study

We performed our initial gene expression analysis using DAVID because it is a widely used tool that, unlike species-specific tools such as WormExp, provides GO terms applicable across all species that in our view facilitates the generation of hypotheses and further studies. A limitation is that the general clusters lack details regarding sub-clusters of genes that may highlight important biological functions. Indeed, our more detailed analysis identified additional pathways that are part of the most highly enriched GO terms. To exclude potential contributions from reproduction, we used L4 animals in our gene expression studies. Although it is possible that different cholesterol concentrations slightly affect larval development, the animals seem to reach adulthood at the same time. Because we performed the survival studies using adults, we do not believe that any potential difference during larval development would account for the survival differences that we have observed.

## Methods

All methods can be found in the accompanying Transparent Methods supplemental file.

## Data and Code Availability

RNA sequencing data were deposited in NCBI GEO database under the accession numbers: GSE136881 and GSE137058.

## Supplemental Information

Document S1. Transparent Methods and Figures S1–S9

Table S1. Upregulated and Downregulated Genes More than 5-Folds (p < 0.05) in Animals Grown on Different Cholesterol Concentrations, Related to Figure 2 The RNA-seq raw data were submitted and assigned Gene Expression Omnibus (GEO) accession numbers: GSE136881 and GSE137058, Related to Figure 2.

Table S2. GO Enrichment Analysis Using DAVID Bioinformatics Database, Related to Figure 2

Table S3. List of Upregulated and Downregulated Immune Genes in Animals Grown on Different Cholesterol Concentrations, Related to Figure 2

Table S4. Representation Factors for Gene Sets among Cholesterol-Dependent Immune Genes using WormExp, Related to Figure 2

Table S5. Representation factors for gene sets among cholesterol-dependent immune genes using a biased approach, Related to Figure 2

Table S6. Primers used in this study, Related to Figures 3, 4, and S7
