# Cholesterol Regulates Innate Immunity via Nuclear Hormone Receptor NHR-8

**DOI:** 10.1016/j.isci.2021.103361

**Published:** 2021-11-02

**Authors:** Benson Otarigho, Alejandro Aballay

## Main text

(iScience *23*, 101068-1–101068-11; May 22, 2020)

In the originally published article, the authors made a mistake in analyzing RNA sequencing (RNA-seq) data corresponding to 5 μg/ml vs. 0 cholesterol presented. Therefore, incorrect annotations were placed in the data corresponding to 5 μg/ml cholesterol as shown in Figures 2A–2D and S4 and their corresponding legends, Tables S1, S2, S3, S4, and S5, and associated text throughout the manuscript. Values were mistakenly presented in the reversed order (i.e., the reported genes were annotated as downregulated instead of upregulated in the absence of cholesterol). These are vital considerations necessary for reporting and reproducibility of data, and the authors apologize for this error. They further confirm that this does not change the scientific message of the paper.

**Figure:**
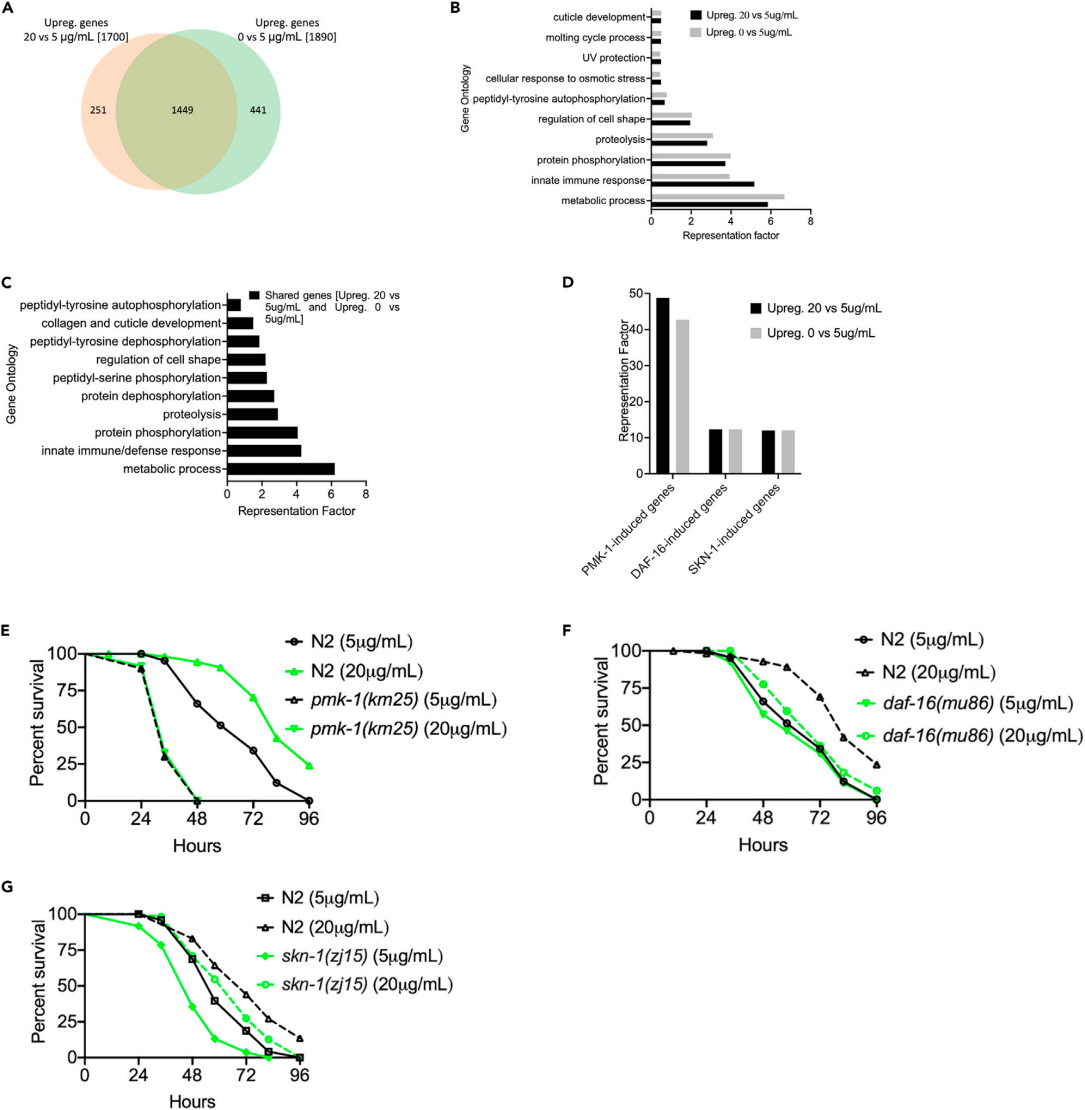
Figure 2. Cholesterol-Mediated Immunity Primarily Acts through a p38/PMK-1 MAPK Pathway

## Supplemental information

Document S1. Transparent Methods and Figures S1–S9

Table S1. Upregulated and Downregulated Genes More than 5-Folds (p < 0.05) in Animals Grown on Different Cholesterol Concentrations, Related to Figure 2 The RNA-seq raw data were submitted and assigned Gene Expression Omnibus (GEO) accession numbers: GSE136881 and GSE137058, Related to Figure 2

Table S2. GO Enrichment Analysis Using DAVID Bioinformatics Database, Related to Figure 2

Table S3. List of Upregulated and Downregulated Immune Genes in Animals Grown on Different Cholesterol Concentrations, Related to Figure 2

Table S4. Representation Factors for Gene Sets among Cholesterol-Dependent Immune Genes using WormExp, Related to Figure 2

Table S5. Representation factors for gene sets among cholesterol-dependent immune genes using a biased approach, Related to Figure 2

