# Supplementary material for: Cholesterol Regulates Innate Immunity via Nuclear Hormone Receptor NHR-8
Source: iScience. 2020 Apr 18;23(5):101068. doi: 10.1016/j.isci.2020.101068 (PMC7195545; doi:10.1016/j.isci.2020.101068)
Supplement: Document S1. Transparent Methods and Figures S1–S9 [file mmc1.pdf]

iScience, Volume *23*

Supplemental Information

Cholesterol Regulates Innate Immunity via Nuclear Hormone Receptor NHR-8

Benson Otarigho and Alejandro Aballay

TRANSPARENT METHODS

Bacterial strains

The bacterial strains used in this study are *Escherichia coli* OP50, *E. coli* HT115(DE3), *Pseudomonas aeruginosa* PA14, and *P. aeruginosa* PA14-GFP. Bacteria were grown in Luria-Bertani (LB) broth at 37°C.

C. elegans strains and growth conditions

Hermaphrodite *C. elegans* (var. Bristol) wild type (N2) was used as the control unless otherwise indicated. The *C. elegans* strains used in this study were CF1038 *daf-16(mu86)*, KU25 *pmk-1(km25), CB75 mec-2(e75)*, RB1645 *c56e6.5(ok2034)*, VC999 *tag-340(ok1496)*, VC2712 *f52f12.7(ok3347)*, RB1919 *W07E6.3(ok2498)*, RB1095 *chup-1(ok1073)*, VC452 *chup-1(gk245),* GR1452 veIs13 *[col-19::GFP + rol-6(su1006)] V. mgEx725 [lin-4::let-7 + ttx-3::*RFP], AA292 *daf-36(k114)*, AE501 *nhr-8(ok186)*, RG1228 *daf-9(rh50)*, DR2281 *daf-9(m540)*, AA1 *daf-12(rh257)*, AA10 *daf-12(rh286)* AA34 *daf-12(rh61)* AA86 *daf-12(rh61rh411)*, MGH171 *alxIs9* [*vha-6p::sid-1::SL2::GFP*], NU3 *dbl-1(nk3)*, KU21 *kgb-1(km21)*, QV225 *skn-1(zj15),* and VC1518 *atf-7(gk715)*, which were obtained from the Caenorhabditis Genetics Center (University of Minnesota, Minneapolis, MN). Other strains that were obtained from National BioResource Project (NBRP) of Japan and used in this work were FX19275 *nhr-8(tm1800)* and FX00599 *ncr-1(tm599)*. The rescued strain; *nhr-8(ok186);Pnhr-8::nhr-8::RFP* was obtained upon request from Anne Lespine of INTHERES, Université de Toulouse, INRA, ENVT, Toulouse, France. The intestine rescued strain, *nhr-8(ok186);Pvha-6::nhr-8* was generated by cloning *nhr-8* DNA downstream *vha-6* promoter. All strains were backcrossed with wild type *C. elegans*.

All strains as well as the control animals were grown at 20ºC on nematode growth medium (NGM) plates seeded with *E. coli* OP50 as the food source (Brenner, 1974). Synchronized Animals were grown from eggs to young adults on plates containing different cholesterol concentrations. The recipe for the control NGM plates was as follows: 3 g/L NaCl, 3 g/L peptone, 20 g/L agar, 5 µg/mL cholesterol (Sigma, grade ≥99%), 1 mM MgSO4, 1 mM $CaCl_2$, and 25 mM potassium phosphate buffer (pH 6.0). NGM plates were also prepared using 0 µg/mL (low) and 20 µg/mL (high) cholesterol. In the 0 µg/mL plates, ethanol alone was added while the other components remained the same. No antibiotics were added to the NGM plates except where indicated. While simply omitting cholesterol from NGM pleases provides sub-optimal growing conditions, the complete removal of even trace amounts of sterols by replacing agar with agarose extracted with organic solvents has a

stronger impact on development as it causes the animals to eventually cease to grow and results in a reduce brood size (Merris et al., 2003), (Matyash et al., 2004). Because this can affect the outcome of survival experiments, we decided to use NGM plates with or without cholesterol supplementation.

RNA interference (RNAi)

Knockdown of targeted genes was achieved using RNAi by feeding the animals with *E. coli* strain HT115(DE3) expressing double-stranded RNA (dsRNA) homologous to a target gene (Fraser et al., 2000; Timmons and Fire, 1998). RNAi was carried out according to (Singh and Aballay, 2017; Sun et al., 2011). Briefly, *E. coli* with the appropriate vectors were grown in LB broth containing ampicillin (100 µg/mL) and tetracycline (12.5 µg/mL) at 37°C overnight and plated onto NGM plates containing 100 µg/mL ampicillin and 3 mM isopropyl β-D-thiogalactoside (IPTG) (RNAi plates). RNAi-expressing bacteria were grown at 37°C for 12-14 hours. Gravid adults were transferred to RNAi-expressing bacterial lawns and allowed to lay eggs for 2-3 hours. The gravid adults were removed, and the eggs were allowed to develop at 20°C to young adults for subsequent assays. The RNAi clones were from the Ahringer RNAi library.

C. elegans* survival assay on *Pseudomonas aeruginosa

The *C. elegans* survival assay were carried out on wild-type *P. aeruginosa* strain PA14 lawns that were incubated at 37°C for 12 hours. The survival assay utilized full-lawn bacteria prepared by pipetting 20 µL of 12-hour-cultured *P. aeruginosa* and spreading them completely on the surface of the 3.5-cm-diameter SK plates. The plates were incubated at 37°C for 12 hours and then cooled to room temperature for at least one hour before seeding with synchronized young adult hermaphroditic animals. The SK plates were prepared using 0 µg/mL (low), 5 µg/mL (control), and 20 µg/mL (high) cholesterol. The killing assays were performed at 25°C, and live animals were transferred daily to fresh plates. Animals were scored at the indicated times for survival and transferred to fresh pathogen lawns each day until no progeny were produced. Animals were considered dead when they failed to respond to touch and no pharyngeal pumping was observed. Each experiment was performed in triplicate (n = 60 animals).

Brood size assay

The brood size assay was performed according to (Kenyon et al., 1993; Singh and Aballay, 2017). Ten L4 animals from egg-synchronized populations were transferred to individual plates with corresponding cholesterol conditions (described above) and incubated at 20°C.

The animals were transferred to fresh plates every 24 hours. The progenies were counted and removed daily.

***C. elegans* longevity assays**

Longevity assays were performed on NGM plates containing live- or heat killed-*E. coli* OP50 plates according to (Sun et al., 2011) with the above-described different concentrations of cholesterol. Animals were scored as alive, dead, or gone each day. Animals that failed to display touch-provoked or pharyngeal movement were scored as dead. Experimental groups contained 60 to 100 animals. The assays were performed at 20°C.

Intestinal bacterial load visualization and quantification

Intestinal bacterial loads were visualized and quantified according to (Singh and Aballay, 2017; Singh and Aballay, 2006; Sun et al., 2011). Briefly, *P. aeruginosa*-GFP lawns were prepared as described above. The plates were cooled to ambient temperature for at least one hour before seeding with young gravid adult hermaphroditic animals, and the setup was placed at 25°C for 24 hours. The animals were transferred from *P. aeruginosa*-GFP plates to the center of fresh *E. coli* plates for 10 minutes to eliminate *P. aeruginosa*-GFP on their body. The step was repeated two times more to further eliminate external *P. aeruginosa*-GFP left from earlier steps. Subsequently, ten animals were collected and used for fluorescence imaging to visualize the bacterial load, while another ten animals were transferred into 100 µL of PBS plus 0.01% Triton X-100 and ground. Serial dilutions of the lysates (10^1-10^{10}) were seeded onto LB plates containing 50 µg/mL of kanamycin to select for *P. aeruginosa*-GFP cells and grown overnight at 37°C. Single colonies were counted the next day and represented as the number of bacterial cells or CFU per animal. Three independent experiments were performed for each condition.

***col-19*::GFP visualization and quantification**

GR1452 animals that express GFP at late L4/young adult transition were synchronized and grown in the absence of cholesterol supplementation, 5, and 20 µg/mL cholesterol. Fluorescence imaging of *col-19*::GFP was done using young adult stage (~65 hours post hatching) animals that were grown at 20°C under the different cholesterol concentrations. The fluorescence intensity was quantified using Image J software.

Fluorescence imaging

Fluorescence imaging was carried out according to (Singh and Aballay, 2017; Singh and Aballay, 2006; Sun et al., 2011) with slight modifications. Briefly, animals were anesthetized using an M9 salt solution containing 50 mM sodium azide and mounted onto 2% agar pads. The animals were then visualized for bacterial load using a Leica M165 FC fluorescence

stereomicroscope. Fluorescence was quantified using Fiji-ImageJ (https://imagej.net/Fiji/Downloads).

RNA sequencing and analyses

Approximately 40 gravid animals were placed for 3 hours on 10-cm NGM plates (seeded with *E. coli* OP50) (described above) to obtain a synchronized population, which developed and grew to L4 larval stage at 20ºC. Animals were washed off the plates with M9, frozen in QIAzol using ethanol/dry ice, and stored at -80 prior to RNA extraction. Total RNA was extracted using the RNeasy Plus Universal Kit (Qiagen, Netherlands). Residual genomic DNA was removed using TURBO DNase (Life Technologies, Carlsbad, CA). A total of 6 µg of total RNA was reverse-transcribed with random primers using the High-Capacity cDNA Reverse Transcription Kit (Applied Biosystems, Foster City, CA).

The library construction and RNA sequencing in the BGISEQ-500 platform was performed according to (Yao et al., 2018; Zhu et al., 2018), and paired-end reads of 100 bp were obtained for subsequent data analysis. The RNA sequence data were analyzed using a workflow constructed for Galaxy (https://usegalaxy.org) (Afgan et al., 2018; Afgan et al., 2016; Amrit and Ghazi, 2017). The RNA reads were aligned to the *C. elegans* genome (WS230) using the aligner STAR. Counts were normalized for sequencing depth and RNA composition across all samples. Differential gene expression analysis was then performed using normalized samples. Genes exhibiting at least a two-fold change were considered differentially expressed. The differentially expressed genes were subjected to SimpleMine tools from Wormbase (https://www.wormbase.org/tools/mine/simplemine.cgi) to generate information such as the wormBase ID and gene names, which were employed for further analyses. Gene ontology analysis was performed using the wormBase IDs in DAVID Bioinformatics Database (https://david.ncifcrf.gov) (Dennis et al., 2003) and a *C. elegans* data enrichment analysis tool (https://wormbase.org/tools/enrichment/tea/tea.cgi). The Venn diagrams were obtained using the web tool InteractiVenn (http://www.interactivenn.net) (Heberle et al., 2015) and bioinformatics and evolutionary genomics tool (http://bioinformatics.psb.ugent.be/webtools/Venn/).

RNA isolation and quantitative reverse transcription-PCR (qRT-PCR)

Animals were synchronized, and total RNA extraction was performed following the above-described protocol. qRT-PCR was conducted using the Applied Biosystems One-Step Real-time PCR protocol using SYBR Green fluorescence (Applied Biosystems) on an Applied Biosystems 7900HT real-time PCR machine in 96-well-plate format. Twenty-five-microliter reactions were analyzed as outlined by the manufacturer (Applied Biosystems). The relative

fold changes of the transcripts were calculated using the comparative $CT(2^{-\Delta\Delta CT})$ method and normalized to pan-actin (*act-1, -3, -4*). The cycle thresholds of the amplification were determined using StepOnePlus™ Real-Time PCR System Software v2.3 (Applied Biosystems). All samples were run in triplicate. The primer sequences are available upon request and are presented in Table S6.

Generation of transgenic *C. elegans*

The *nhr-8* DNA was amplified from genomic DNA of Bristol N2 *C. elegans* adult worms as template using the primers presented in Table S6. Linearization of plasmid pPD95.77_Pvha-6_SL2 was performed by restriction digestion using SalI and SmaI enzymes. The amplified nhr-8 DNA was cloned under the vha-6 promoter in plasmid pPD95.77_*Pvha-6*_SL2 between the SalI and SmaI sites to generate the expression clone pPD95.77_*Pvha-6_nhr-8*_SL2. The construct was purified and sequenced. Young adult hermaphrodite *nhr-8(ok186) C. elegans* were transformed by microinjection of plasmids into the gonads according to (Mello and Fire, 1995; Mello et al., 1991). A mixture containing the pPD95.77_*Pvha-6_nhr-8*_SL2 plasmids (40 ng/µL) and pRF4_ *rol-6(su1006)* (10 ng/µL) as a transformation marker was injected into the worm. Successful transformation was determined by identification of the selection marker as dominant roller. At least three independent lines carrying extra chromosomal arrays were obtained for each construct. Only worms with dominant roller were selected for further experiments.

Western blot analysis

Whole-worm lysates of L4 stage grown at 5 and 20 µg/mL cholesterol were prepared according to (Cao and Aballay, 2016). Phosphorylated PMK-1 protein was detected using an anti-active p38 polyclonal antibody from rabbit (1:2000, Promega) and β-actin was detected using a monoclonal anti-actin antibody from mouse (1:1000, Abcam).

Quantification and statistical analysis

Statistical analysis was performed with Prism 8 version 8.1.2 (GraphPad). All error bars represent the standard deviation (SD). The two-sample t test was used when needed, and the data were judged to be statistically significant when $p < 0.05$. In the figures, asterisks (*) denote statistical significance as follows: *, $p < 0.05$, **, $p < 0.001$, ***, $p < 0.0001$, as compared with the appropriate controls. The Kaplan-Meier method was used to calculate the survival fractions, and statistical significance between survival curves was determined using the log-rank test. All experiments were performed in triplicate.

Supplemental References

Afgan, E., Baker, D., Batut, B., Van Den Beek, M., Bouvier, D., Čech, M., Chilton, J., Clements, D., Coraor, N., and Grüning, B.A. (2018). The Galaxy platform for accessible, reproducible and collaborative biomedical analyses: 2018 update. Nucleic acids research *46*, W537-W544.

Afgan, E., Baker, D., Van den Beek, M., Blankenberg, D., Bouvier, D., Čech, M., Chilton, J., Clements, D., Coraor, N., and Eberhard, C. (2016). The Galaxy platform for accessible, reproducible and collaborative biomedical analyses: 2016 update. Nucleic acids research *44*, W3-W10.

Amrit, F.R., and Ghazi, A. (2017). Transcriptomic Analysis of C. elegans RNA Sequencing Data Through the Tuxedo Suite on the Galaxy Project. JoVE (Journal of Visualized Experiments), e55473.

Cao, X., and Aballay, A. (2016). Neural inhibition of dopaminergic signaling enhances immunity in a cell-non-autonomous manner. Current Biology *26*, 2329-2334.

Dennis, G., Sherman, B.T., Hosack, D.A., Yang, J., Gao, W., Lane, H.C., and Lempicki, R.A. (2003). DAVID: database for annotation, visualization, and integrated discovery. Genome biology *4*, R60.

Fraser, A.G., Kamath, R.S., Zipperlen, P., Martinez-Campos, M., Sohrmann, M., and Ahringer, J. (2000). Functional genomic analysis of *C. elegans* chromosome I by systematic RNA interference. Nature *408*, 325.

Heberle, H., Meirelles, G.V., da Silva, F.R., Telles, G.P., and Minghim, R. (2015). InteractiVenn: a web-based tool for the analysis of sets through Venn diagrams. BMC bioinformatics *16*, 169.

Kenyon, C., Chang, J., Gensch, E., Rudner, A., and Tabtiang, R. (1993). A *C. elegans* mutant that lives twice as long as wild type. Nature *366*, 461.

Matyash, V., Entchev, E.V., Mende, F., Wilsch-Bräuninger, M., Thiele, C., Schmidt, A.W., Knölker, H.-J., Ward, S., and Kurzchalia, T.V. (2004). Sterol-derived hormone (s) controls entry into diapause in *Caenorhabditis elegans* by consecutive activation of DAF-12 and DAF-16. PLoS biology *2*, e280.

Mello, C., and Fire, A. (1995). DNA transformation. In Methods in cell biology (Elsevier), pp. 451-482.

Mello, C.C., Kramer, J.M., Stinchcomb, D., and Ambros, V. (1991). Efficient gene transfer in C. elegans: extrachromosomal maintenance and integration of transforming sequences. The EMBO journal *10*, 3959-3970.

Merris, M., Wadsworth, W.G., Khamrai, U., Bittman, R., Chitwood, D.J., and Lenard, J. (2003). Sterol effects and sites of sterol accumulation in *Caenorhabditis elegans* developmental requirement for 4α-methyl sterols. Journal of lipid research *44*, 172-181.

Singh, J., and Aballay, A. (2017). Endoplasmic reticulum stress caused by lipoprotein accumulation suppresses immunity against bacterial pathogens and contributes to immunosenescence. MBio *8*, e00778-00717.

Singh, V., and Aballay, A. (2006). Heat-shock transcription factor (HSF)-1 pathway required for *Caenorhabditis elegans* immunity. Proceedings of the National Academy of Sciences *103*, 13092-13097.

Sun, J., Singh, V., Kajino-Sakamoto, R., and Aballay, A. (2011). Neuronal GPCR controls innate immunity by regulating noncanonical unfolded protein response genes. Science *332*, 729-732.

Timmons, L., and Fire, A. (1998). Specific interference by ingested dsRNA. Nature *395*, 854.

Yao, L., Wang, J., Li, B., Meng, Y., Ma, X., Si, E., Ren, P., Yang, K., Shang, X., and Wang, H. (2018). Transcriptome sequencing and comparative analysis of differentially-expressed isoforms in the roots of Halogeton glomeratus under salt stress. Gene *646*, 159-168.

Zhu, F.-Y., Chen, M.-X., Ye, N.-H., Qiao, W.-M., Gao, B., Law, W.-K., Tian, Y., Zhang, D., Zhang, D., and Liu, T.-Y. (2018). Comparative performance of the BGISEQ-500 and Illumina HiSeq4000 sequencing platforms for transcriptome analysis in plants. Plant methods *14*, 69.

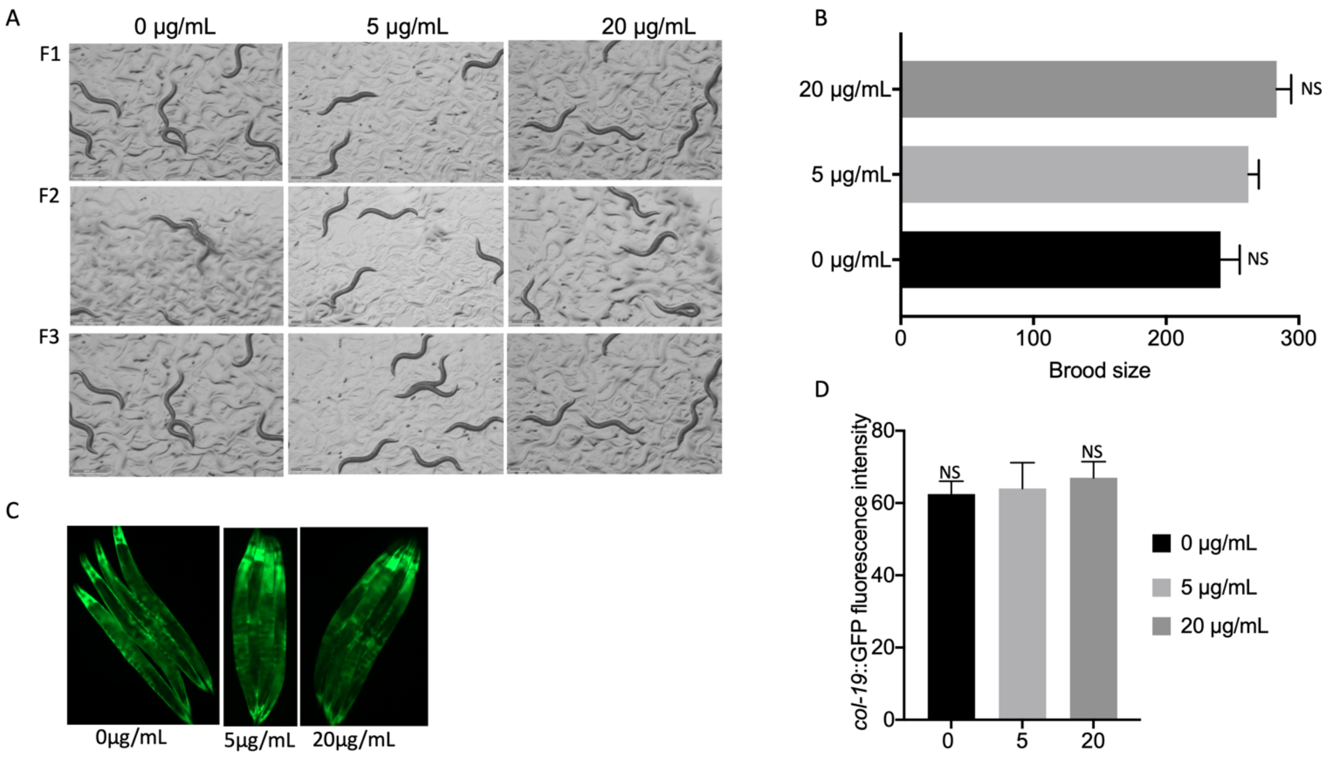

Figure S1. The absence of cholesterol supplementation does not affect the development or brood size of *C. elegans.* Related to Figure 1.
(A) Microscopic images of animals grown in the absence and different cholesterol supplementation concentrations for three generations. Microscopic images were obtained at the young adult stage (~65 hours post hatching).
(B) Brood size of animals grown in the absence and different cholesterol concentrations. Bars represent means while error bars indicate SD; P = NS.
(C) Fluorescence images of GR1452 young adult animals (expressing GFP) grown in the absence of cholesterol supplementation and at different cholesterol concentrations. Microscopic images were obtained at the young adult stage (~65 hours post hatching).
(D) Quantification of *col-19*::GFP expression of GR1452 animals grown in the absence of cholesterol supplementation and at different cholesterol concentrations. Fluorescence was quantified using Fiji-ImageJ. Bars represent means while error bars indicate SD; P = NS.

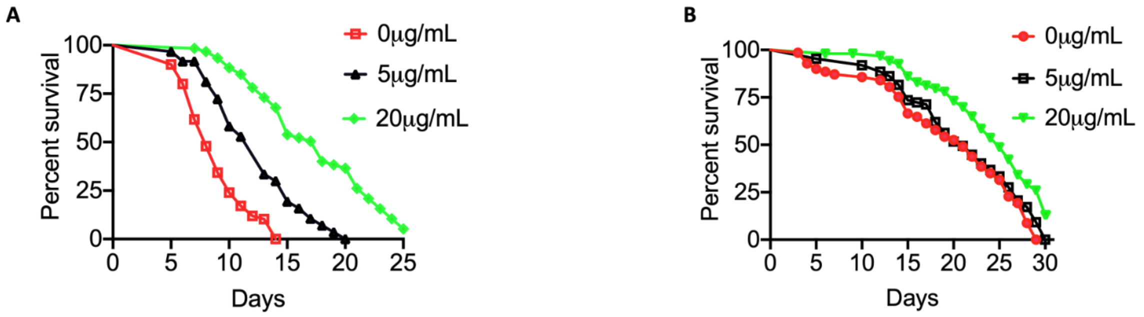

Figure S2. Lifespan of the *C elegans* grown on different cholesterol concentrations on live and heat-killed *E. coli*. Related to Figure 1.

(A) Survival of WT animals grown on *E. coli* at different cholesterol concentrations, transferred to live *E. coli* cultured at the control cholesterol concentration (5 µg/mL), and scored for survival. WT animals on live *E. coli* 5 µg/mL vs. 0 µg/mL, $P < 0.0001$; 20 µg/mL, $P < 0.0001$.

(B) Survival of WT animals grown on *E. coli* at different cholesterol concentrations, transferred to heat-killed *E. coli* seeded on plates with control cholesterol concentration (5 µg/mL) and 50 µg/mL streptomycin, and scored for survival. WT animals on heat-killed *E. coli* 5 µg/mL vs. 0 µg/mL, P = NS; 20 µg/mL, $P < 0.001$.

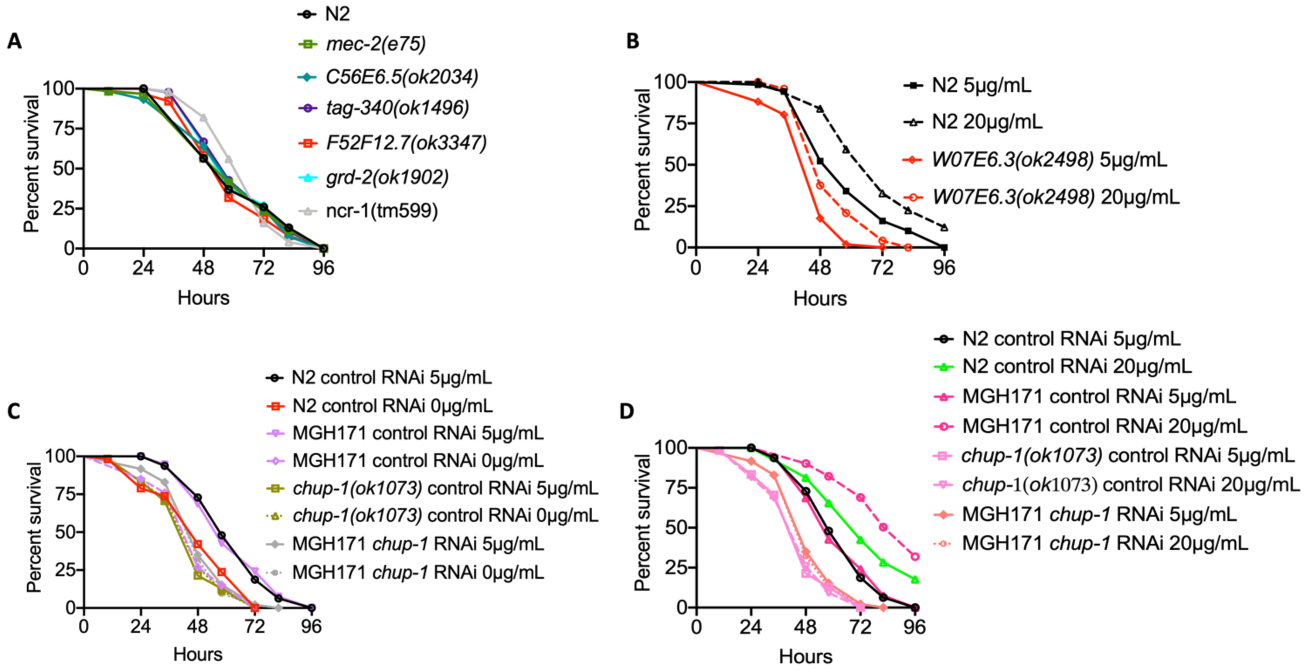

Figure S3. Survival of animals deficient in cholesterol transporters infected with *P. aeruginosa*. Related to Figure 1.

(A) WT animals and mutants in cholesterol transporters were grown on 5µg/mL cholesterol, exposed to *P. aeruginosa,* and scored for survival. WT animal vs. cholesterol transporter loss-of-function animals*,* P = NS.

(B) WT animals and *W07E6.3(ok2498)* were grown on 5 and 20 µg/mL cholesterol, exposed to *P. aeruginosa*, and scored for survival. *W07E6.3(ok2498)* grown on 5µg/mL cholesterol vs. 20 µg/mL cholesterol, P < 0.001

(C) Control, MGH171(*chup-1* RNAi), WT (*chup-1* RNAi), and *chup-1(ok1073)* RNAi animals were grown on were 0 and 5µg/mL cholesterol, exposed to *P. aeruginosa,* and scored for survival. MGH171 *chup-1* RNAi grown on 5µg/mL cholesterol vs. MGH171 *chup-1* RNAi grown on 0µg/mL, P = NS.

(D) Control, MGH171(*chup-1* RNAi), WT (*chup-1* RNAi), and *chup-1(ok1073)* RNAi animals were grown on were 20 and 5µg/mL cholesterol, exposed to *P. aeruginosa,* and scored for survival. MGH171 *chup-1* RNAi grown on 5µg/mL cholesterol vs. MGH171 *chup-1* RNAi grown on 20µg/mL, P = NS.

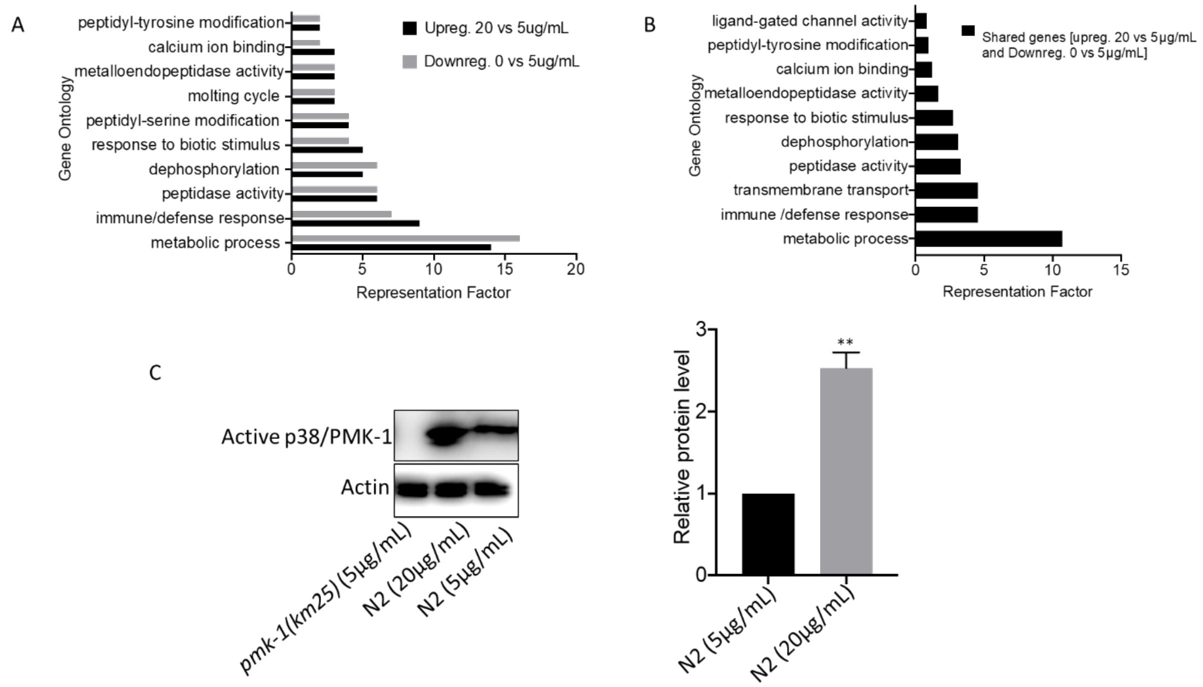

Figure S4: Analysis of cholesterol genes and active PMK-1/p38 levels. Related to Figures 2 and 4.

(A) Enrichment analysis of upregulated genes at 20 vs 5 µg/mL and downregulated genes at 0 vs 5 µg/mL cholesterol. The cutoff is based on the filtering thresholds of $P < 0.05$ and arranged according to the representation factor.

(B) Enrichment analysis of shared genes between animals grown at 20 vs. 5 µg/mL and 0 vs. 5 µg/mL cholesterol. The cutoff is based on the filtering thresholds of $P < 0.05$ and arranged according to the representation factor.

(C) Western blot analysis of active PMK-1/p38 levels in wild-type animals grown at different cholesterol concentration. Image quantification was performed using the software program Fiji/ImageJ. Bars represent means while error bar indicates SD; $^{**}p < 0.001$

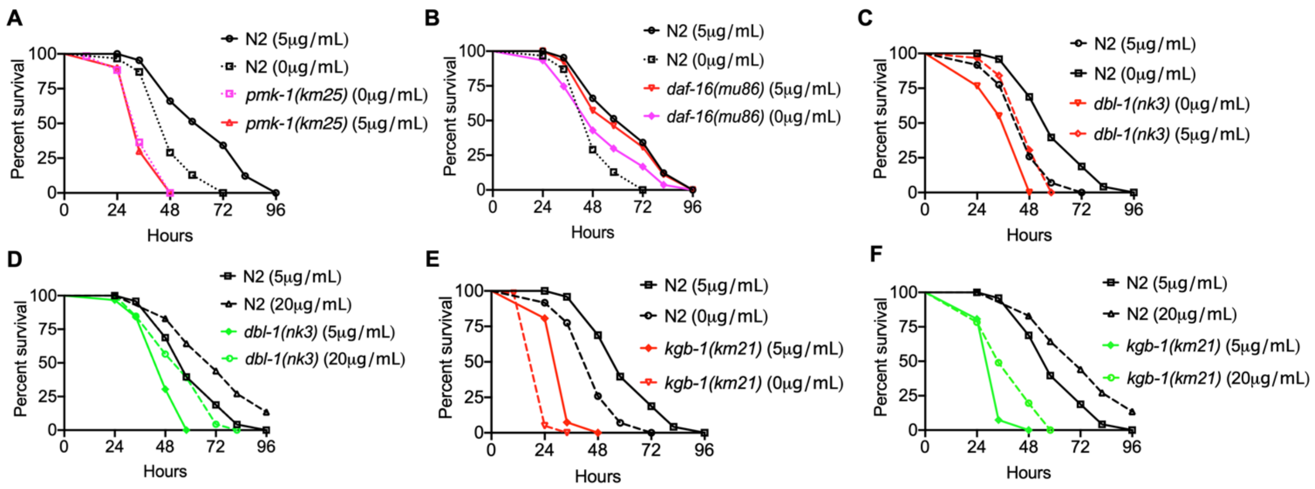

Figure S5. Survival of mutants in immune genes grown at different cholesterol concentrations. Related to Figure 2.

(A) WT and *pmk-1(km25)* animals were grown on 0 and 5 µg/mL cholesterol, exposed to *P. aeruginosa,* and scored for survival. WT animals grown on 5µg/mL cholesterol (control) vs. 0µg/mL, P< 0.0001; *pmk-1(km25)* 0µg/mL, P< 0.0001; *pmk-1(km25)* 5µg/mL, P= P>0.0001. *pmk-1(km25)* 0µg/mL vs. *pmk-1(km25)* 5µg/mL, P=NS.

(B) WT and *daf-16(mu86)* animals were grown on 0 and 5 µg/mL cholesterol, exposed to *P. aeruginosa,* and scored for survival. WT animals grown on 5µg/mL cholesterol (control) vs. 0µg/mL, P< 0.0001; *daf-16(mu86)* 0µg/mL, P< 0.0001; *daf-16(mu86)* 5µg/mL, P= NS.

(C) WT and *dbl-1(nk3)* animals were grown on 0 and 5µg/mL cholesterol, exposed to *P. aeruginosa,* and scored for survival. *dbl-1(nk3)* animals grown on 5 µg/mL cholesterol (control) vs. 0 µg/mL, P< 0.001.

(D) WT and *dbl-1(nk3)* animals were grown on 20 and 5µg/mL cholesterol, exposed to *P. aeruginosa,* and scored for survival. *dbl-1(nk3)* animals grown on 5µg/mL cholesterol (control) vs. 20, P< 0.001.

(E) WT and *kgb-1(km21)* animals were grown on 0 and 5µg/mL cholesterol, exposed to *P. aeruginosa,* and scored for survival. *kgb-1(km21)* animals grown on 5µg/mL cholesterol (control) vs. 0, P< 0.001.

(F) WT and *kgb-1(km21)* animals were grown on 20 and 5µg/mL cholesterol, exposed to *P. aeruginosa,* and scored for survival. *kgb-1(km21)* animals grown on 5µg/mL cholesterol (control) vs. 20, P< 0.001.

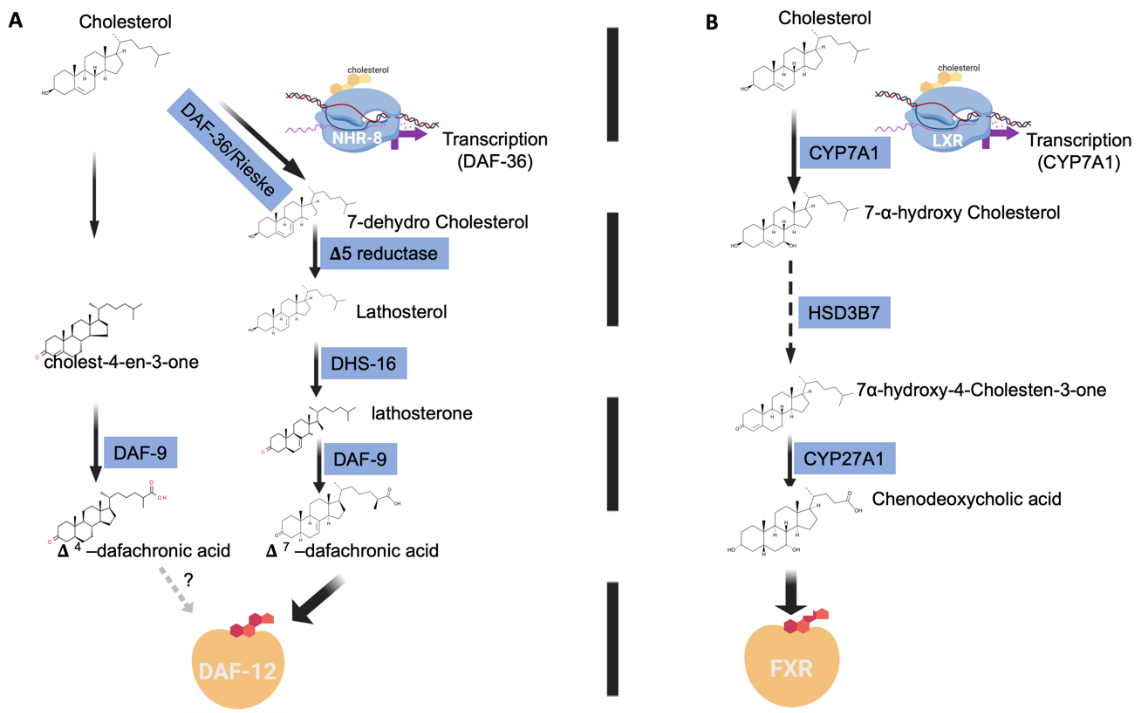

Figure S6. Diagram of the steroid biosynthesis pathways. Related to Figure 3. (A) *C. elegans* and (B) mammalian steps and genes are shown purple color. Modified from: Antebi, A., 2015. Nuclear receptor signal transduction in *C. elegans*. WormBook, 1, p.49.

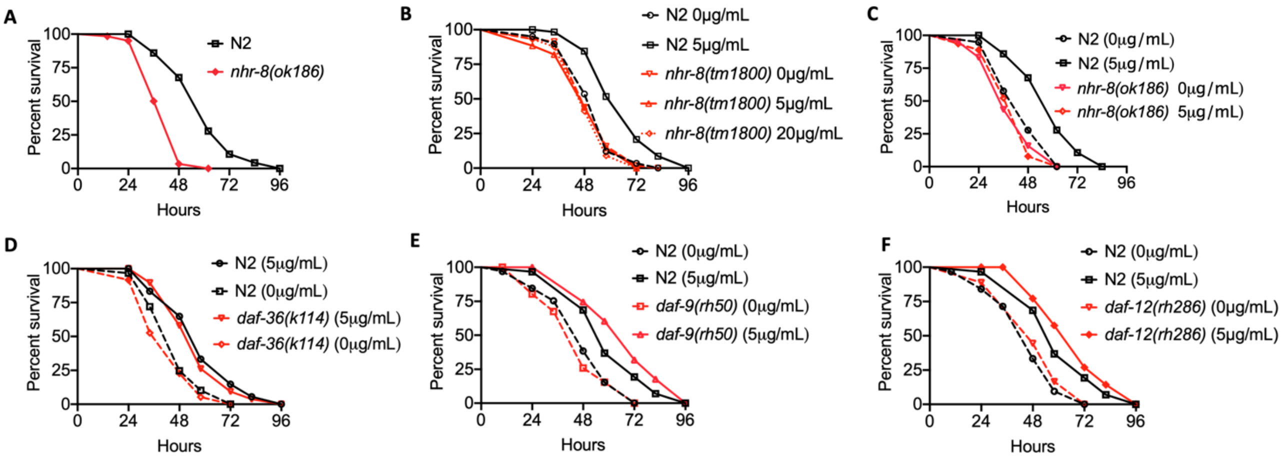

Figure S7. Survival of mutants in genes required for steroid synthesis grown at different cholesterol concentrations. Related to Figure 3

(A) WT and *nhr-8(ok186)* animals were grown on 5 µg/mL cholesterol, exposed to *P. aeruginosa,* and scored for survival. WT animals vs. *nhr-8(ok186)*, P < 0.0001.

(B) WT and *nhr-8(tm1800)* animals were grown on 0, 5 and 20 µg/mL cholesterol supplementation, exposed to *P. aeruginosa,* and scored for survival. WT animals vs. *nhr-8(tm1800)*, P < 0.001; *8(tm1800)* at 5 µg/mL vs. *8(tm1800)* at 0 µg/mL, P = NS; vs *8(tm1800)* at 20 µg/mL, P= NS.

(C) WT and *nhr-8(ok186)* animals were grown on 0 and 5µg/mL cholesterol, exposed to *P. aeruginosa,* and scored for survival. WT animals grown on 5µg/mL cholesterol (control) vs. WT animals 0µg/mL, P< 0.0001; *nhr-8(ok186)* 0µg/mL, P< 0.0001; *nhr-8(ok186)* 5µg/mL,

(D) WT and *daf-36(k114)* animals were grown on 0 and 5µg/mL cholesterol, exposed to *P. aeruginosa,* and scored for survival. WT animals grown on 5µg/mL cholesterol (control) vs. WT animals 0µg/mL, P< 0.0001; *daf-36(k114)* 0µg/mL, P< 0.0001; *daf-36(k114)* 5µg/mL, P = NS. *daf-36(k114)* animals on 0µg/mL vs. *daf-36(k114)* 5µg/mL, P< 0.0001.

(E) WT and *daf-9(rh50)* animals were grown on 0 and 5µg/mL cholesterol, exposed to *P. aeruginosa,* and scored for survival. WT animals grown on 5µg/mL cholesterol (control) vs. WT animals 0µg/mL, P< 0.0001; *daf-9(rh50)* 0µg/mL, P< 0.0001; *daf-9(rh50)* 5µg/mL, P < 0.001. *daf-9(rh50)* animals on 0µg/mL vs. *daf-9(rh50)* 5µg/mL, P< 0.0001.
P< 0.0001. *nhr-8(ok186)* animals on 0µg/mL vs. *nhr-8(ok186)* 5µg/mL, P=NS.

(F) WT and *daf-12(rh286)* animals were grown on 0 and 5µg/mL cholesterol, exposed to *P. aeruginosa,* and scored for survival. WT animals grown on 5µg/mL cholesterol (control) vs. WT animals on 0µg/mL, P< 0.0001; *daf-12(rh286)* 0µg/mL, P< 0.0001; *daf-12(rh286)* 5µg/mL, P< 0.001. *daf-12(rh286)* mutant on 0µg/mL vs. *daf-12(rh286)* 5µg/mL, P< 0.0001.

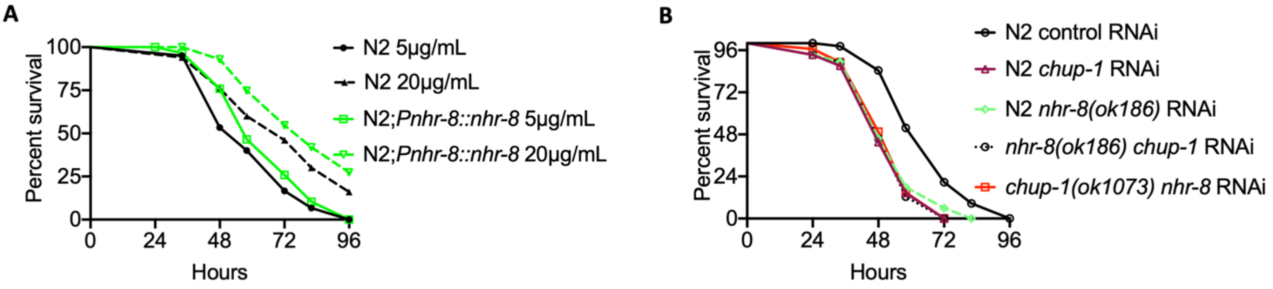

Figure S8: NHR-8 and CHUP-1 are part of the same pathway that promotes cholesterol-mediated innate immunity . Related to Figure 3

(A) WT;*Pnhr-8::nhr-8* animals were grown in the absence of cholesterol supplementation and at 5µg/mL cholesterol, exposed to *P. aeruginosa,* and scored for survival. WT animals grown on 20µg/mL cholesterol (control) vs. WT;*Pnhr-8::nhr-8* at 20µg/mL, P<0.001.

(B) Control RNAi on WT, *chup-1(ok1013),* and *nhr-8(186)* alongside with *chup-1* RNAi on *nhr-8(186)* animals were grown on 5 µg/mL cholesterol, exposed to *P. aeruginosa*, and scored for survival. *nhr-8(186)* (chup-1 RNAi) vs. *chup-1(ok1013)*(control RNAi), P=NS; vs. *nhr-8(186)* (control RNAi), P=NS.

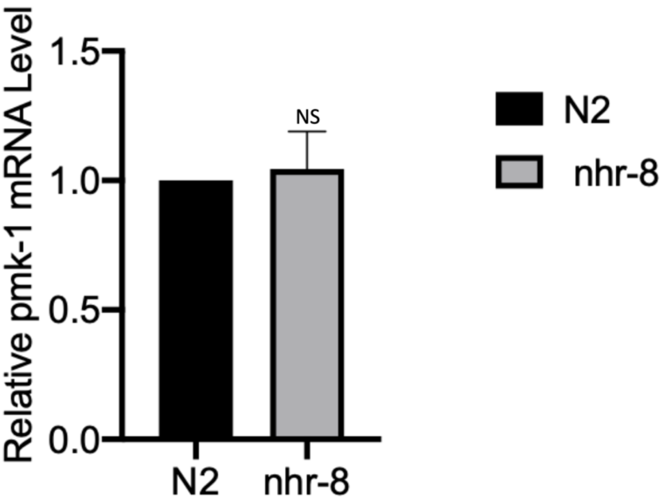

Figure S9. ***pmk-1*** **expression in N2 and** ***nhr-8(ok186)*** **animals. Related to Figure 4.**
Gene expression of *pmk-1* in *nhr-8(ok186)* and N2 animals grown on 5 µg/mL cholesterol on *E. coli*. Bars represent means while error bar indicates SD; P=NS
