# Supplementary material for: Cholesterol Regulates Innate Immunity via Nuclear Hormone Receptor NHR-8
Source: iScience. 2020 Apr 18;23(5):101068. doi: 10.1016/j.isci.2020.101068 (PMC7195545; doi:10.1016/j.isci.2020.101068)
Supplement: Table S6. Primers used in this study, Related to Figures 3, 4, and S7 [file mmc7.docx]

| **Table S7. Primers for quantitative reverse transcription-PCR** | | | |
| --- | --- | --- | --- |
| **Gene name** | **Forward primer sequence (5'-3')** | | **Reverse primer sequence (5’-3’)** |
| *Pan-act* | TCGGTATGGGACAGAAGGAC | | CATCCCAGTTGGTGACGATA |
| *F55G11.8* | TAGTTTCGGCTACGGGAAATG | | TCCATGAAGCTGGCCAATAA |
| *C17H12.6* | GCCCGTGTTAGATTGGAGAA | | GAACTGATGGAGAACGACTTGA |
| *C05A9.1* | TATTGGATGAAGCGACGAGTG | | GTGCAACAACAACAGTGGATAG |
| *Irg-1* | GGATGATCTTGTTCCGTACCC | | CCTCTCCAGTTTCGTTCATCTT |
| *Irg-2* | GGGTGTGATGGTAGCAGAAA | | AGCTGATGTAAATGACGGATAGG |
| *Lys-6* | GGCTTGATCTCTCTGCCTTT | | TACCTCTACTACCGGGAACTATG |
|  |  | |  |
|  |  | |  |
| **Primer for generation of transgenic animal** | | | |
|  | **Forward primer sequence (5'-3')** | **Reverse primer sequence (5’-3’)** | |
| *nhr-8* | CTAAAACCTAGTCGAATACAAACTATTGCGCCCTTTC | CCTTTGGCCAATCCCATGTGCAATAATTCATGGTTAATAA | |
|  |  |  | |
|  |  |  | |
