# Supplementary material for: Cholesterol Regulates Innate Immunity via Nuclear Hormone Receptor NHR-8
Source: iScience. 2021 Nov 2;24(11):103361. doi: 10.1016/j.isci.2021.103361 (PMC8571716; doi:10.1016/j.isci.2021.103361)
Supplement: Document S1. Transparent Methods and Figures S1–S9 [file mmc1.pdf]

iScience, Volume *24*

Supplemental information

Cholesterol Regulates Innate Immunity via Nuclear Hormone Receptor NHR-8

Benson Otarigho and Alejandro Aballay

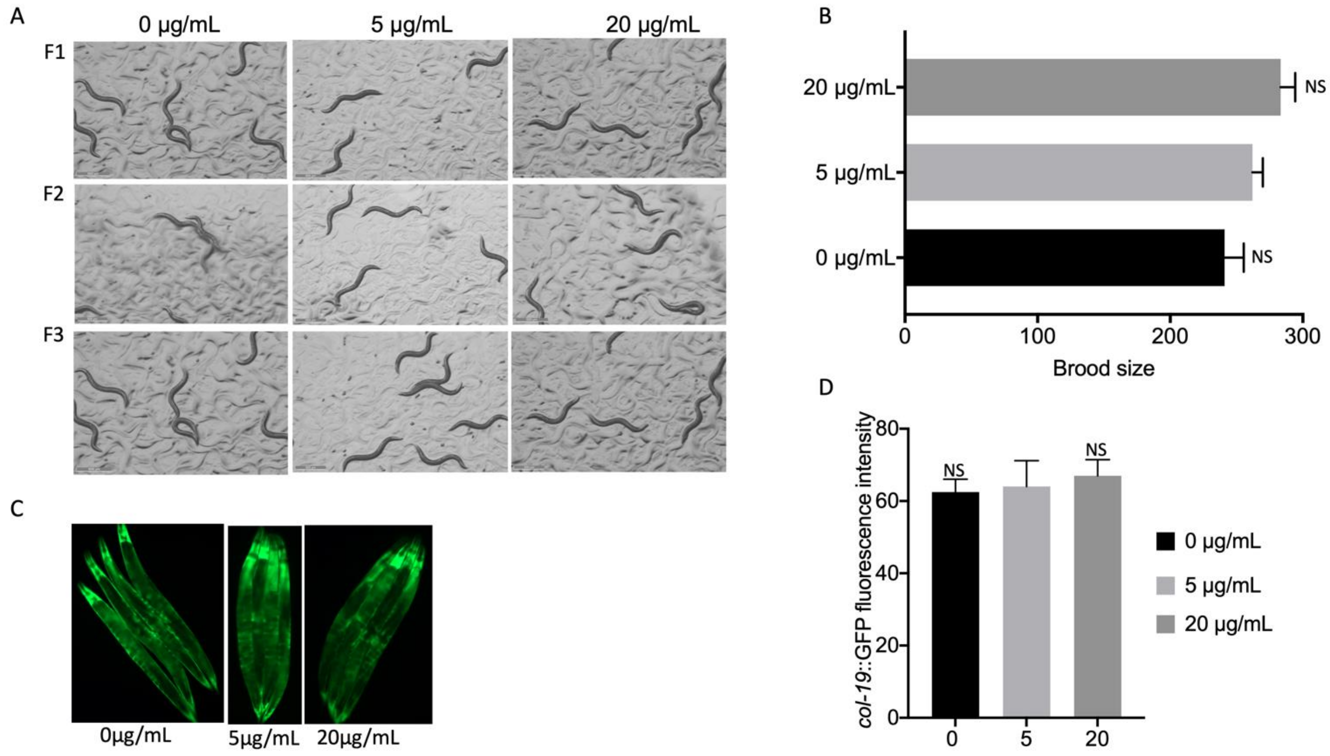

Figure S1. The absence of cholesterol supplementation does not affect the development or brood size of *C. elegans.* Related to Figure 1.

(A) Microscopic images of animals grown in the absence and different cholesterol supplementation concentrations for three generations. Microscopic images were obtained at the young adult stage (~65 hours post hatching).

(B) Brood size of animals grown in the absence and different cholesterol concentrations. Bars represent means while error bars indicate SD; P = NS.

(C) Fluorescence images of GR1452 young adult animals (expressing GFP) grown in the absence of cholesterol supplementation and at different cholesterol concentrations. Microscopic images were obtained at the young adult stage (~65 hours post hatching).

(D) Quantification of *col-19*::GFP expression of GR1452 animals grown in the absence of cholesterol supplementation and at different cholesterol concentrations. Fluorescence was quantified using Fiji-ImageJ. Bars represent means while error bars indicate SD; P = NS.

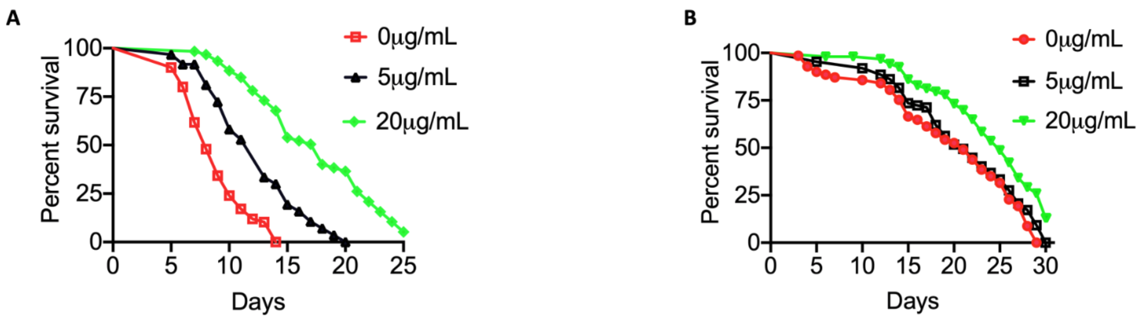

Figure S2. Lifespan of the *C elegans* grown on different cholesterol concentrations on live and heat-killed *E. coli*. Related to Figure 1.

(A) Survival of WT animals grown on *E. coli* at different cholesterol concentrations, transferred to live *E. coli* cultured at the control cholesterol concentration (5 μg/mL), and scored for survival. WT animals on live *E. coli* 5 μg/mL vs. 0 μg/mL, $P < 0.0001$; 20 μg/mL, $P < 0.0001$.

(B) Survival of WT animals grown on *E. coli* at different cholesterol concentrations, transferred to heat-killed *E. coli* seeded on plates with control cholesterol concentration (5 μg/mL) and 50 μg/mL streptomycin, and scored for survival. WT animals on heat-killed *E. coli* 5 μg/mL vs. 0 μg/mL, P = NS; 20 μg/mL, $P < 0.001$.

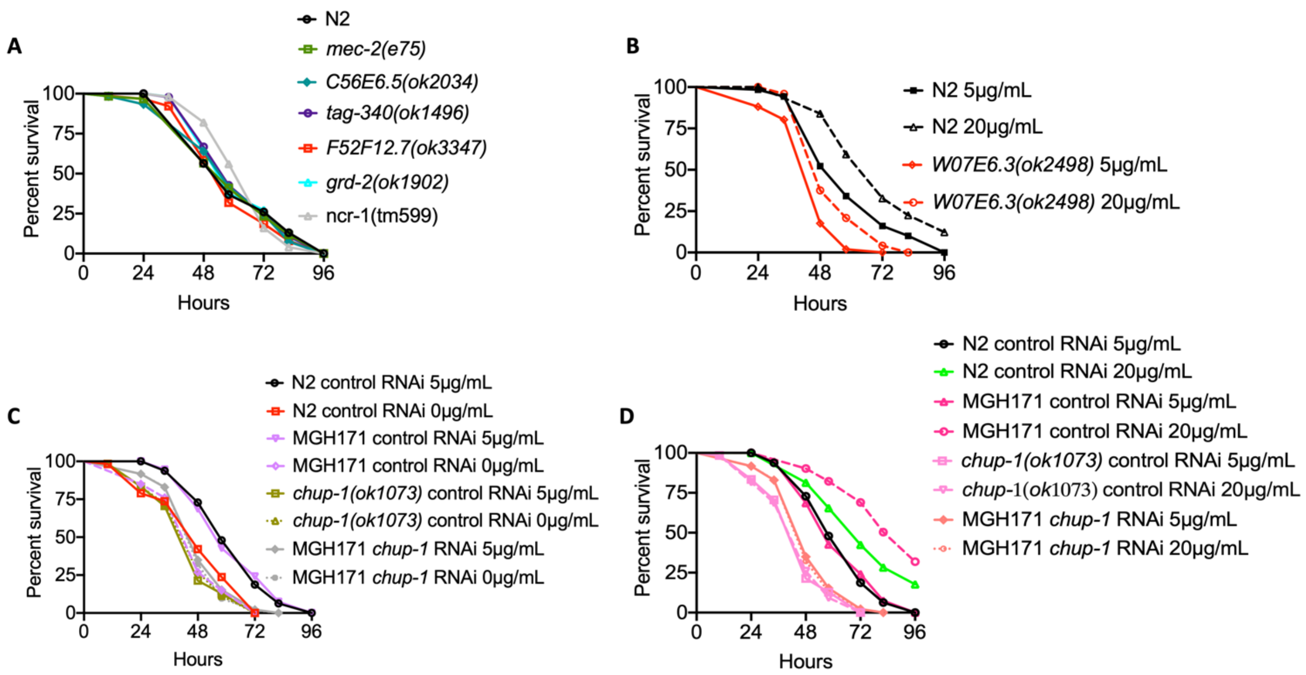

Figure S3. Survival of animals deficient in cholesterol transporters infected with *P. aeruginosa*. Related to Figure 1.

(A) WT animals and mutants in cholesterol transporters were grown on 5µg/mL cholesterol, exposed to *P. aeruginosa*, and scored for survival. WT animal vs. cholesterol transporter loss-of-function animals, P = NS.

(B) WT animals and *W07E6.3(ok2498)* were grown on 5 and 20 µg/mL cholesterol, exposed to *P. aeruginosa*, and scored for survival. *W07E6.3(ok2498)* grown on 5µg/mL cholesterol vs. 20 µg/mL cholesterol, $P < 0.001$

(C) Control, MGH171(*chup-1* RNAi), WT (*chup-1* RNAi), and *chup-1(ok1073)* RNAi animals were grown on were 0 and 5µg/mL cholesterol, exposed to *P. aeruginosa*, and scored for survival. MGH171 *chup-1* RNAi grown on 5µg/mL cholesterol vs. MGH171 *chup-1* RNAi grown on 0µg/mL, P = NS.

(D) Control, MGH171(*chup-1* RNAi), WT (*chup-1* RNAi), and *chup-1(ok1073)* RNAi animals were grown on were 20 and 5µg/mL cholesterol, exposed to *P. aeruginosa*, and scored for survival. MGH171 *chup-1* RNAi grown on 5µg/mL cholesterol vs. MGH171 *chup-1* RNAi grown on 20µg/mL, P = NS.

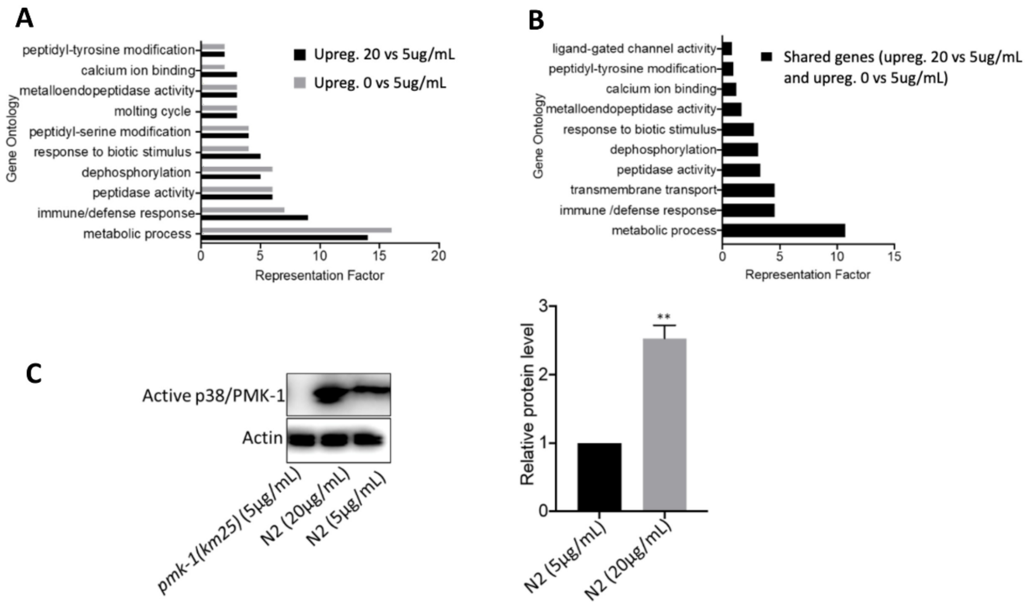

Figure S4: Analysis of cholesterol genes and active PMK-1/p38 levels. Related to Figures 2 and 4.

(A) Enrichment analysis of upregulated genes at 20 vs 5 µg/mL and at 0 vs 5 µg/mL cholesterol. The cutoff is based on the filtering thresholds of $P < 0.05$ and arranged according to the representation factor.

(B) Enrichment analysis of shared genes between animals grown at 20 vs. 5 µg/mL and 0 vs. 5 µg/mL cholesterol. The cutoff is based on the filtering thresholds of $P < 0.05$ and arranged according to the representation factor.

(C) Western blot analysis of active PMK-1/p38 levels in wild-type animals grown at different cholesterol concentration. Image quantification was performed using the software program Fiji/ImageJ. Bars represent means while error bar indicates SD; **$p < 0.001$

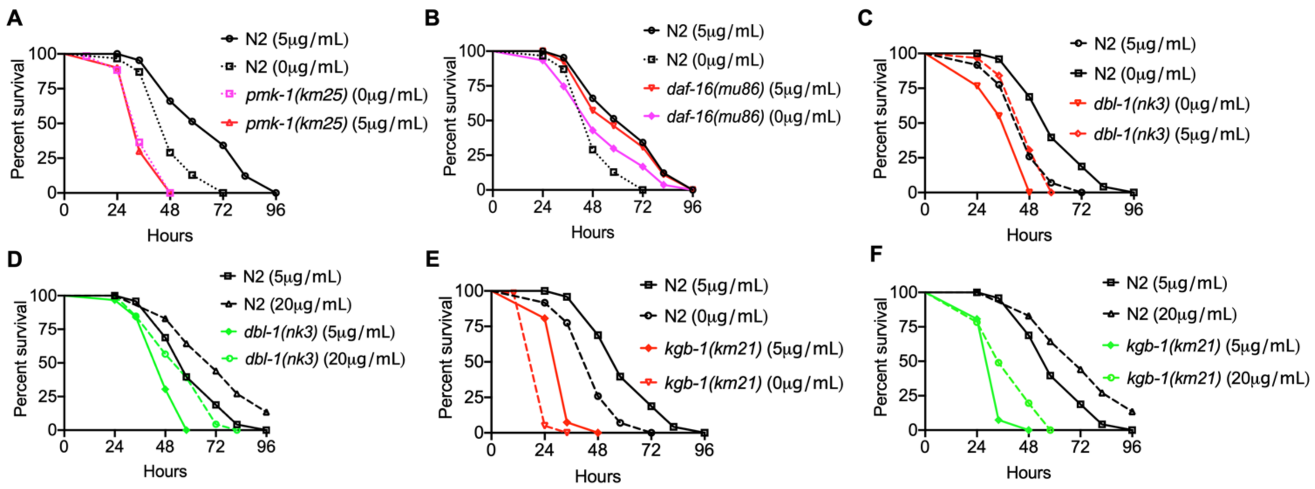

Figure S5. Survival of mutants in immune genes grown at different cholesterol concentrations. Related to Figure 2.

(A) WT and *pmk-1(km25)* animals were grown on 0 and 5 µg/mL cholesterol, exposed to *P. aeruginosa*, and scored for survival. WT animals grown on 5µg/mL cholesterol (control) vs. 0µg/mL, P< 0.0001; *pmk-1(km25)* 0µg/mL, P< 0.0001; *pmk-1(km25)* 5µg/mL, P= P>0.0001. *pmk-1(km25)* 0µg/mL vs. *pmk-1(km25)* 5µg/mL, P=NS.

(B) WT and *daf-16(mu86)* animals were grown on 0 and 5 µg/mL cholesterol, exposed to *P. aeruginosa*, and scored for survival. WT animals grown on 5µg/mL cholesterol (control) vs. 0µg/mL, P< 0.0001; *daf-16(mu86)* 0µg/mL, P< 0.0001; *daf-16(mu86)* 5µg/mL, P= NS.

(C) WT and *dbl-1(nk3)* animals were grown on 0 and 5µg/mL cholesterol, exposed to *P. aeruginosa*, and scored for survival. *dbl-1(nk3)* animals grown on 5 µg/mL cholesterol (control) vs. 0 µg/mL, P< 0.001.

(D) WT and *dbl-1(nk3)* animals were grown on 20 and 5µg/mL cholesterol, exposed to *P. aeruginosa*, and scored for survival. *dbl-1(nk3)* animals grown on 5µg/mL cholesterol (control) vs. 20, P< 0.001.

(E) WT and *kgb-1(km21)* animals were grown on 0 and 5µg/mL cholesterol, exposed to *P. aeruginosa*, and scored for survival. *kgb-1(km21)* animals grown on 5µg/mL cholesterol (control) vs. 0, P< 0.001.

(F) WT and *kgb-1(km21)* animals were grown on 20 and 5µg/mL cholesterol, exposed to *P. aeruginosa*, and scored for survival. *kgb-1(km21)* animals grown on 5µg/mL cholesterol (control) vs. 20, P< 0.001.

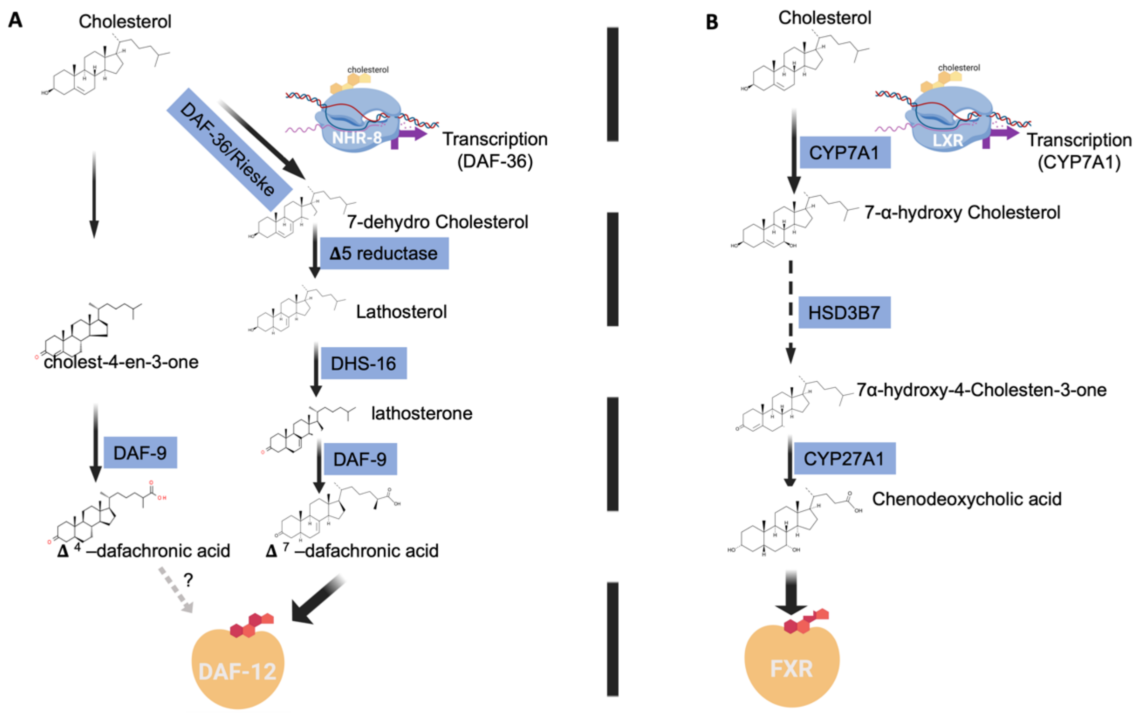

Figure S6. Diagram of the steroid biosynthesis pathways. Related to Figure 3.
(A) *C. elegans* and (B) mammalian steps and genes are shown purple color.
Modified from: Antebi, A., 2015. Nuclear receptor signal transduction in *C. elegans*. WormBook, 1, p.49.

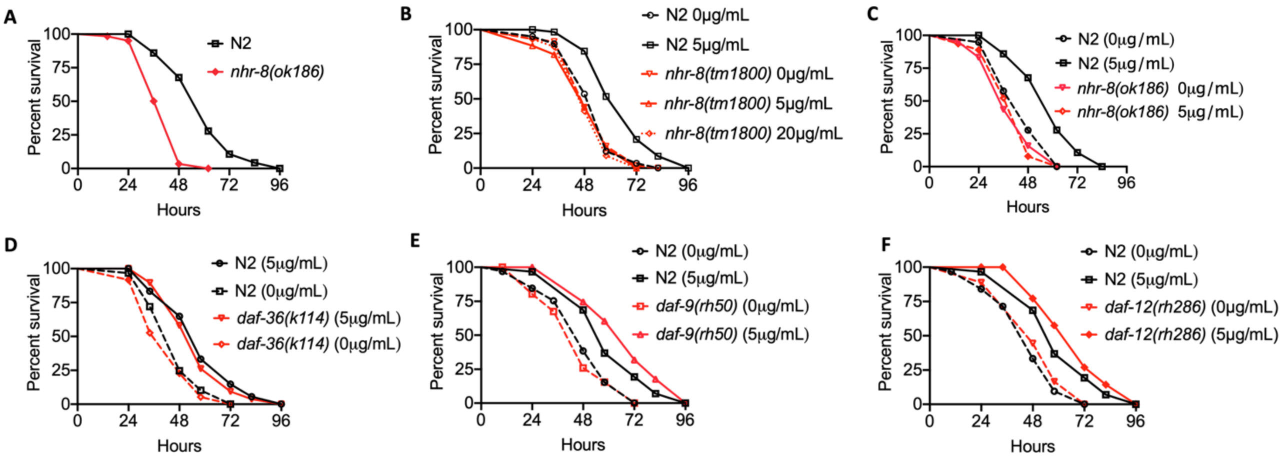

Figure S7. Survival of mutants in genes required for steroid synthesis grown at different cholesterol concentrations. Related to Figure 3

(A) WT and *nhr-8(ok186)* animals were grown on 5 µg/mL cholesterol, exposed to *P. aeruginosa*, and scored for survival. WT animals vs. *nhr-8(ok186)*, P < 0.0001.

(B) WT and *nhr-8(tm1800)* animals were grown on 0, 5 and 20 µg/mL cholesterol supplementation, exposed to *P. aeruginosa*, and scored for survival. WT animals vs. *nhr-8(tm1800)*, P < 0.001; *8(tm1800)* at 5 µg/mL vs. *8(tm1800)* at 0 µg/mL, P = NS; vs *8(tm1800)* at 20 µg/mL, P= NS.

(C) WT and *nhr-8(ok186)* animals were grown on 0 and 5µg/mL cholesterol, exposed to *P. aeruginosa*, and scored for survival. WT animals grown on 5µg/mL cholesterol (control) vs. WT animals 0µg/mL, P< 0.0001; *nhr-8(ok186)* 0µg/mL, P< 0.0001; *nhr-8(ok186)* 5µg/mL,

(D) WT and *daf-36(k114)* animals were grown on 0 and 5µg/mL cholesterol, exposed to *P. aeruginosa*, and scored for survival. WT animals grown on 5µg/mL cholesterol (control) vs. WT animals 0µg/mL, P< 0.0001; *daf-36(k114)* 0µg/mL, P< 0.0001; *daf-36(k114)* 5µg/mL, P = NS. *daf-36(k114)* animals on 0µg/mL vs. *daf-36(k114)* 5µg/mL, P< 0.0001.

(E) WT and *daf-9(rh50)* animals were grown on 0 and 5µg/mL cholesterol, exposed to *P. aeruginosa*, and scored for survival. WT animals grown on 5µg/mL cholesterol (control) vs. WT animals 0µg/mL, P< 0.0001; *daf-9(rh50)* 0µg/mL, P< 0.0001; *daf-9(rh50)* 5µg/mL, P < 0.001. *daf-9(rh50)* animals on 0µg/mL vs. *daf-9(rh50)* 5µg/mL, P< 0.0001.
P< 0.0001. *nhr-8(ok186)* animals on 0µg/mL vs. *nhr-8(ok186)* 5µg/mL, P=NS.

(F) WT and *daf-12(rh286)* animals were grown on 0 and 5µg/mL cholesterol, exposed to *P. aeruginosa*, and scored for survival. WT animals grown on 5µg/mL cholesterol (control) vs. WT animals on 0µg/mL, P< 0.0001; *daf-12(rh286)* 0µg/mL, P< 0.0001; *daf-12(rh286)* 5µg/mL, P< 0.001. *daf-12(rh286)* mutant on 0µg/mL vs. *daf-12(rh286)* 5µg/mL, P< 0.0001.

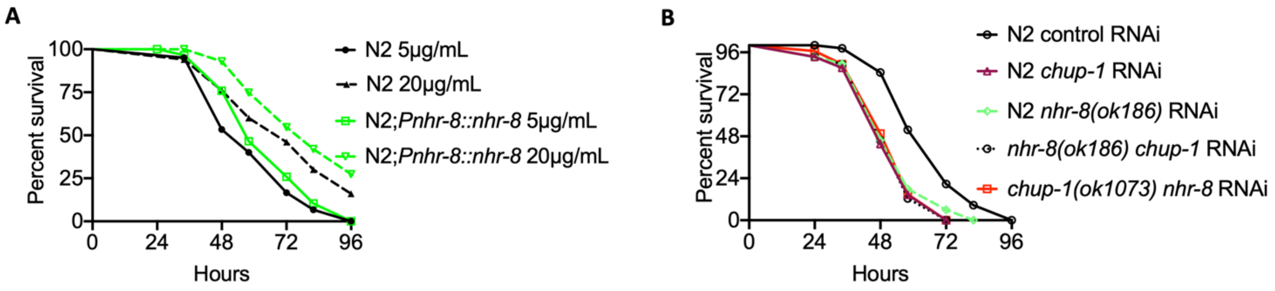

Figure S8: NHR-8 and CHUP-1 are part of the same pathway that promotes cholesterol-mediated innate immunity . Related to Figure 3

(A) WT;*Pnhr-8::nhr-8* animals were grown in the absence of cholesterol supplementation and at 5µg/mL cholesterol, exposed to *P. aeruginosa*, and scored for survival. WT animals grown on 20µg/mL cholesterol (control) vs. WT;*Pnhr-8::nhr-8* at 20µg/mL, P<0.001.

(B) Control RNAi on WT, *chup-1(ok1013)*, and *nhr-8(186)* alongside with *chup-1* RNAi on *nhr-8(186)* animals were grown on 5 µg/mL cholesterol, exposed to *P. aeruginosa*, and scored for survival. *nhr-8(186)* (chup-1 RNAi) vs. *chup-1(ok1013)*(control RNAi), P=NS; vs. *nhr-8(186)* (control RNAi), P=NS.

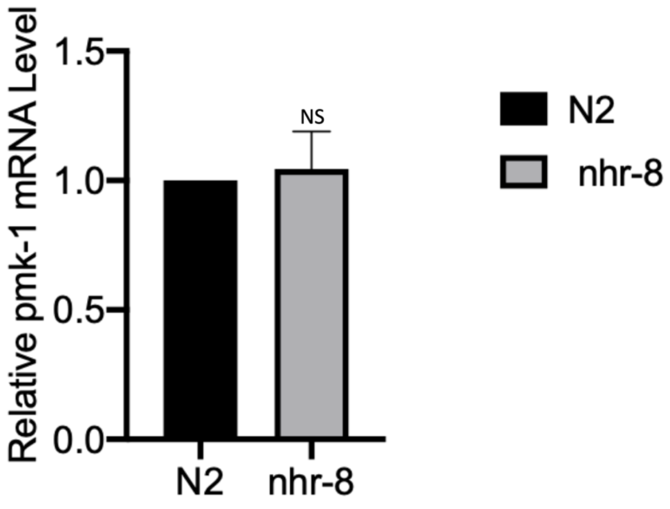

Figure S9. *pmk-1* expression in N2 and *nhr-8(ok186)* animals. Related to Figure 4.
Gene expression of *pmk-1* in *nhr-8(ok186)* and N2 animals grown on 5 µg/mL cholesterol on *E. coli*. Bars represent means while error bar indicates SD; P=NS
